# Genome-Wide Investigation and Expression Profiling Under Abiotic Stresses of a Soybean Unknown Function (DUF21) and Cystathionine-β-Synthase (CBS) Domain-Containing Protein Family

**DOI:** 10.1007/s10528-020-09991-w

**Published:** 2020-08-10

**Authors:** Qingnan Hao, Yanyan Yang, Zhihui Shan, Haifeng Chen, Chanjuan Zhang, Limiao Chen, Songli Yuan, Xiaojuan Zhang, Shuilian Chen, Zhonglu Yang, Dezhen Qiu, Xinan Zhou

**Affiliations:** 1 Key Laboratory of Oil Crop Biology, Ministry of Agriculture, Wuhan, 430062 China; 2 Oil Crops Research Institute of Chinese Academy of Agriculture Sciences, Wuhan, China

**Keywords:** Soybean, CBS domain, CBSDUF genes, Abiotic stress

## Abstract

Cystathionine-β-synthase (CBS) domain-containing proteins (CDCPs) constitute a large family in plants, and members of this family have been implicated in a variety of biological processes. However, the precise functions and the underlying mechanisms of most members of this family in plants remain to be elucidated. CBSDUF proteins belong to the CDCP superfamily, which contains one domain of unknown function (DUF21) and an N terminus that is adjacent to two intracellular CBS domains. In this study, a comprehensive genome database analysis of soybean was performed to investigate the role(s) of these CBSDUFs and to explore their nomenclature, classification, chromosomal distribution, exon–intron organization, protein structure, and phylogenetic relationships; the analysis identified a total of 18 putative CBSDUF genes. Using specific protein domains and phylogenetic analysis, the CBSDUF gene family was subdivided into eight groups. The soybean CBSDUF genes showed an uneven distribution on 12 chromosomes of *Glycine max*. RNA-seq transcriptome data from different tissues in public databases revealed tissue-specific and differential expression profiles of the GmCBSDUFs, and qPCR analysis revealed that certain groups of soybean CBSDUFs are likely involved in specific stress responses. In addition, GmCBSDUF3 transgenic Arabidopsis was subjected to phenotypic analysis under NaCl, PEG, and ABA stress treatments. The overexpression of GmCBSDUF3 could enhance tolerance to drought and salt stress in Arabidopsis. This study presents a first comprehensive look at soybean CBSDUF proteins and provides valuable resources for functionally elucidating this protein subgroup within the CBS domain-containing protein family.

**Electronic supplementary material:**

The online version of this article (10.1007/s10528-020-09991-w) contains supplementary material, which is available to authorized users.

## Introduction

Expression profiling studies in different organisms have suggested that proteins with unknown functions play important roles in many biological processes (Gollery et al. 2006). These proteins have been divided into two types: one includes proteins with obscure features that lack defined motifs or domains (POFs) and the other includes proteins with defined features that contain at least one previously defined domain or motif (PDFs). Among the latter, a group of proteins containing the cystathionine-β-synthase (CBS) domain might play important roles in stress response/tolerance in Arabidopsis under various stress conditions (Kushwaha et al. 2009). Since the CBS domain was first identified in the *Archaebacterium Methanococcus jannaschii* (Bateman 1997), CDCPs have been found to represent a large superfamily of evolutionarily conserved proteins. Kushwaha et al. identified CDCPs in whole-genome analyses of *Oryza sativa* and *Arabidopsis thaliana* and found that the CBS domain coexists with other functional domain(s) in most of these proteins, which may indicate their probable functions. Based on whether they have additional domain(s), these proteins were further classified into different subclasses: CBSX, CBSCLC, CBSSIS, CBSPPR, CBSIMPDH, CBSCBS, CBSCBSPB and CBSDUF. These subclasses possess various functions, including cytoplasmic targeting, subcellular localization of chloride channels (CLC), protein–protein interaction, protein regulation, sensing of cellular energy status, and maintenance of intracellular ion gradients (Bateman 1997). For example, the highly conserved structure of CBS domains from CLC plays a role in regulating the common gate (Estevez et al. 2004). AKINbc, a CDCP containing four CBS domains, contributes to SnRK1 heterotrimeric complexes and interacts with two proteins implicated in plant pathogen resistance (Gissot et al. 2006). OsCBSX4, a CDCP, could improve abiotic stress tolerance in plants (Singh et al. 2012). OsBi1, a CDCP, could be induced by BPH and is related to resistance to brown plant hopper in rice plants (Wang et al. 2004). OsCBSX3, a CDCP, is involved in rice resistance to *M. oryzae* (Singh et al. 2012).

However, very few studies have been reported on the CBSDUF subgroup. The CBSDUF subgroup protein contains one domain of unknown function (DUF21) (PF01595) and an N terminus that is adjacent to two intracellular CBS domains. This transmembrane region has no known function. Many of the sequences in this family are annotated as hemolysins because of their similarity to Q54318 (HLYC_BRAHO), which does not contain this domain. Therefore, the functions of DUF21 are still unknown. DUF21 often exists together with CBS domains and plays important roles in plant growth and development. The characteristics of the CBSDUFs in this subgroup are not yet clear. In our previous study, we identified CDCPs in soybean, but there was no detailed analysis of the CBSDUF subgroup. We found that overexpression of soybean GmCBS21, which belongs to the CBSDUF subgroup, possesses a novel function to improve low nitrogen tolerance in *A. thaliana* in our previous study (Hao et al. 2016). In addition, Sinharoy et al. found that a protein containing the CBS-DUF21 domain from *Medicago truncatula* is required for rhizobial infection and symbiotic nitrogen fixation (Sinharoy and Liu 2016). Therefore, considering the above studies, we speculate that proteins in the CBSDUF subgroup may play an important role in regulating biotic and abiotic stress, especially in legumes, and are worthy of further exploration. Soybean is one of the most important oil crops in the world and provides a large proportion of the protein used by humans and animals (Kereszt et al. 2007). However, to date, few data (Hao et al. 2016) are available about proteins in the CBSDUF subgroup in soybean. In this study, we took advantage of bioinformatics and publicly available data to identify and analyze soybean CBSDUF genes on a genome-wide scale. A total of 18 CBSDUFs were identified, and their phylogenetic relationships, gene structures, protein structures, conserved motifs, and expression patterns were analyzed in detail. Furthermore, the expression of CBSDUFs in response to various abiotic stresses as well as low nitrogen treatments in a low N-tolerant soybean variety (Pohuang) was determined. Our results provide a basis for further investigation of the evolution and functions of CBSDUFs.

## Results

### Identification and Phylogenetic Analysis of the Soybean DUF21- and CBS-Domain-Containing Proteins

Eighteen putative GmCBSDUF members were found in the NCBI database and used as queries to conduct BLAST searches against the public genome database (https://phytozome.jgi.doe.gov/pz/portal.html#). If more than one transcript existed, the primary transcript was selected as a representative. Using the same approach, 8, 10, 10, 4, 9, 4, and 4 putative CBSDUF members were identified from common bean (*Phaseolus vulgaris*), *M. truncatula*, *Lotus japonicus*, sorghum, Arabidopsis, rice, and maize, respectively. Table 1 shows the information of CBSDUF genes. Based on available information in the Phytozome 12 database, functional annotations for soybean CBSDUFs were obtained. Less information about the functions of the CBSDUF genes was found. The main functional annotations showed that most of the CBSDUF genes were predicted to be ancient conserved domain protein-related, metal transporter CNNM, or hemolysin-related. The specific functions of these genes remain to be discovered.

**Table 1:** CBSDUFs gene information

Gene name	Locus ID	Protein			Chromosome	Location	Transmembrane helices	Subcellular localization	Annotation
		Length	MW (kDa)	PI					
GmCBSDUF1	Glyma.02G298200	666	74.51	5.06	Gm02	47546918..47555795	4	plas	CBS domain, Transporter associated domain-containing protein
GmCBSDUF2	Glyma.04G032100	487	53.16	6.68	Gm04	2573559..2577999	3	plas	Predicted membrane protein, contains two CBS domains, metal transporter CNNM
GmCBSDUF3	Glyma.06G032200	487	52.99	6.18	Gm06	2493404..2497832	3	plas	Predicted membrane protein, contains two CBS domains, metal transporter CNNM
GmCBSDUF4	Glyma.07G185800	425	47.20	5.58	Gm07	35333504..35340145	3	plas	Predicted membrane protein, contains two CBS domains, metal transporter CNNM
GmCBSDUF5	Glyma.07G256500	493	53.43	6.06	Gm07	43249966..43256033	3	plas	Predicted membrane protein, contains two CBS domains, metal transporter CNNM
GmCBSDUF6	Glyma.08G063400	425	47.27	5.58	Gm08	4867449..4872679	3	plas	Predicted membrane protein, contains two CBS domains, metal transporter CNNM
GmCBSDUF7	Glyma.09G080900	324	36.27	8.60	Gm09	9361547..9364561	3	plas	Ancient conserved domain protein-related, metal transporter CNNM
GmCBSDUF8	Glyma.09G129700	470	52.12	6.03	Gm09	32436191..32440635	3	nucl	Predicted membrane protein, contains two CBS domains, metal transporter CNNM
GmCBSDUF9	Glyma.09G129900	353	39.16	8.81	Gm09	32448316..32450988	3	cyto	Ancient conserved domain protein-related, metal transporter CNNM
GmCBSDUF10	Glyma.13G252800	340	38.20	5.16	Gm13	35918076..35921466	2	cysk	Predicted membrane protein, contains two CBS domains, metal transporter CNNM
GmCBSDUF11	Glyma.13G252900	303	34.10	5.97	Gm13	35923689..35926084	1	cyto	Predicted membrane protein, contains two CBS domains
GmCBSDUF12	Glyma.14G015600	681	76.11	5.18	Gm14	1115792..1125565	5	plas	Hemolysin-related
GmCBSDUF13	Glyma.15G061900	413	45.83	6.36	Gm15	4770579..4777519	3	plas	Predicted membrane protein, contains two CBS domains, metal transporter CNNM
GmCBSDUF14	Glyma.15G062100	423	47.20	5.88	Gm15	4778675..4782856	3	cyto	Predicted membrane protein, contains two CBS domains, metal transporter CNNM
GmCBSDUF15	Glyma.15G103700	489	53.23	5.92	Gm15	8080974..8087923	3	plas	Predicted membrane protein, contains two CBS domains, metal transporter CNNM
GmCBSDUF16	Glyma.16G177500	478	52.94	6.07	Gm16	33867232..33871770	3	cyto	Predicted membrane protein, contains two CBS domains, metal transporter CNNM
GmCBSDUF17	Glyma.17G017700	493	53.59	5.94	Gm17	1340535..1360280	3	plas	Predicted membrane protein, contains two CBS domains, metal transporter CNNM
GmCBSDUF18	Glyma.19G154200	477	52.47	5.67	Gm19	41458397..41464715	3	plas	Predicted membrane protein, contains two CBS domains, metal transporter CNNM
AtCBSDUF1	AT1G03270	499	54.66	6.44	At01	799191..802436	3	chlo	CBS domain-containing protein with a domain of unknown function (DUF21)
AtCBSDUF2	AT1G47330	527	57.93	5.96	At01	17351050..17353875	3	plas	CBS domain-containing protein with a domain of unknown function (DUF21)
AtCBSDUF3	AT1G55930	653	72.93	5.46	At01	20918717..20922232	5	plas	CBS domain-containing protein/transporter associated domain-containing protein
AtCBSDUF4	AT2G14520	423	47.31	5.42	At02	6182193..6184648	3	cyto	CBS domain-containing protein with a domain of unknown function (DUF21)
AtCBSDUF5	AT3G13070	661	73.75	5.00	At03	4191351..4195112	4	plas	CBS domain-containing protein/transporter associated domain-containing protein
AtCBSDUF6	AT4G14230	495	53.49	6.10	At04	8200667..8203238	3	chlo	CBS domain-containing protein with a domain of unknown function (DUF21)
AtCBSDUF7	AT4G14240	494	53.58	5.62	At04	8204347..8207408	3	plas	CBS domain-containing protein with a domain of unknown function (DUF21)
AtCBSDUF8	AT4G33700	424	47.11	5.73	At04	16176276..16179481	3	E.R	CBS domain-containing protein with a domain of unknown function (DUF21)
AtCBSDUF9	AT5G52790	500	55.13	5.93	At05	21391717..21394359	3	cyto	CBS domain-containing protein with a domain of unknown function (DUF21)
PvCBSDUF1	Phvul.001G149200	472	51.81	6.12	Pv01	40258284..40264333	3	plas	Predicted membrane protein, contains two CBS domains, metal transporter CNNM
PvCBSDUF2	Phvul.002G217200	425	47.15	5.20	Pv02	37976948..37982698	3	plas	Predicted membrane protein, contains two CBS domains, metal transporter CNNM
PvCBSDUF3	Phvul.003G093400	491	53.43	6.00	Pv03	19286055..19291575	3	plas	Predicted membrane protein, contains two CBS domains, metal transporter CNNM
PvCBSDUF4	Phvul.004G106300	478	53.14	6.04	Pv04	34726864..34731785	3	cyto	Predicted membrane protein, contains two CBS domains, metal transporter CNNM
PvCBSDUF5	Phvul.004G106600	464	51.90	5.83	Pv04	34753790..34757273	3	nucl	Predicted membrane protein, contains two CBS domains, metal transporter CNNM
PvCBSDUF6	Phvul.006G197700	425	47.53	5.76	Pv06	30311179..30314938	3	E.R	Predicted membrane protein, contains two CBS domains, metal transporter CNNM
PvCBSDUF7	Phvul.008G276000	664	74.04	5.00	Pv08	58474461..58482457	5	plas	Hemolysin-related
PvCBSDUF8	Phvul.009G057700	489	53.20	6.37	Pv09	10511994..10518944	3	plas	Predicted membrane protein, contains two CBS domains, metal transporter CNNM
MtCBSDUF1	Medtr2g010520	429	47.79	5.40	Mt02	2422876..2426201	3	plas	magnesium and cobalt efflux protein CorC, putative
MtCBSDUF2	Medtr3g111830	492	53.62	6.87	Mt03	52319771..52324915	3	cyto	Predicted membrane protein, contains two CBS domains, metal transporter CNNM
MtCBSDUF3	Medtr4g092610	425	47.41	5.22	Mt04	36721936..36727374	3	cyto	Predicted membrane protein, contains two CBS domains, metal transporter CNNM
MtCBSDUF4	Medtr4g117360	492	53.61	5.73	Mt04	48666950..48672872	3	plas	DUF21 domain plant protein
MtCBSDUF5	Medtr5g094740	821	91.73	5.85	Mt05	41394774..41403459	4	plas	CBS domain protein/transporter associated domain protein
MtCBSDUF6	Medtr6g045467	468	51.13	5.30	Mt06	16387634..16392931	3	plas	DUF21 domain plant protein
MtCBSDUF7	Medtr6g051860	423	47.51	6.55	Mt06	18052428..18058032	4	plas	DUF21 domain plant protein
MtCBSDUF8	Medtr6g052300	476	53.04	5.95	Mt06	18250241..18256861	3	cyto	CBS domain protein
MtCBSDUF9	Medtr7g010900	494	54.54	5.72	Mt07	2792470..2796635	3	chlo	DUF21 domain plant protein
MtCBSDUF10	Medtr7g094620	478	53.00	5.98	Mt07	37716843..37721563	4	chlo	DUF21 domain plant protein
OsCBSDUF1	LOC_Os03g03430	518	56.48	7.25	Os03	1476335..1483361	3	plas	Predicted membrane protein, contains two CBS domains, metal transporter CNNM
OsCBSDUF2	LOC_Os03g39640	679	73.80	4.90	Os03	22016151..22029740	4	chlo	Predicted membrane protein, contains two CBS domains
OsCBSDUF3	LOC_Os03g47120	420	46.40	5.49	Os03	26651598..26657847	3	plas	Predicted membrane protein, contains two CBS domains, metal transporter CNNM
OsCBSDUF4	LOC_Os05g32850	528	56.73	5.83	Os05	19232846..19240430	3	plas	Predicted membrane protein, contains two CBS domains, metal transporter CNNM
SbCBSDUF1	Sobic.001G139900	422	46.64	5.44	Sb01	11127471..11132935	3	plas	Predicted membrane protein, contains two CBS domains, metal transporter CNNM
SbCBSDUF2	Sobic.001G176700	678	72.77	5.01	Sb01	14864942..14875714	3	chlo	Hemolysin-related
SbCBSDUF3	Sobic.001G524000	520	56.30	6.95	Sb01	78868430..78873113	3	cyto	Similar to CBS domain-containing protein, putative, expressed
SbCBSDUF4	Sobic.009G128500	518	55.33	5.73	Sb09	48200028..48209242	5	plas	Predicted membrane protein, contains two CBS domains, metal transporter CNNM
ZmCBSDUF1	GRMZM2G045892	522	55.79	5.83	Zm02	144849851..144860479	5	plas	Predicted membrane protein, contains two CBS domains, metal transporter CNNM
ZmCBSDUF2	GRMZM2G050684	422	46.74	5.37	Zm02	257265276..257275848	3	plas	Predicted membrane protein, contains two CBS domains, metal transporter CNNM
ZmCBSDUF3	GRMZM2G092281	520	56.52	6.95	Zm02	153880422..153886707	3	E.R	Metal transporter CNNM
ZmCBSDUF4	GRMZM2G176558	521	56.64	7.21	Zm02	5428401..5433218	3	plas	Metal transporter CNNM
LjCBSDUF1	Lj0g3v0112359	225	25.10	8.90	Lj0g	48894980..48895537	0	chlo	CBS domain-containing protein/transporter associated domain-containing protein
LjCBSDUF2	Lj0g3v0303929	246	26.58	6.07	Lj0g	158331223..158331316	2	vacu	CBS domain-containing protein with a domain of unknown function (DUF21)
LjCBSDUF3	Lj1g3v0270450	294	32.09	5.68	Lj1g	3278636..3278873	3	cyto	CBS domain-containing protein with a domain of unknown function (DUF21)
LjCBSDUF4	Lj1g3v4419930	294	32.09	5.68	Lj1g	51517807..51518044	3	cyto	CBS domain-containing protein with a domain of unknown function
LjCBSDUF5	Lj2g3v3248950	419	46.10	9.00	Lj2g	41604264..41604872	4	plas	CBS domain-containing protein/transporter associated domain-containing protein
LjCBSDUF6	Lj4g3v0412710	425	47.56	5.24	Lj4g	5408952..5409129	3	plas	CBS domain-containing protein with a domain of unknown function (DUF21)
LjCBSDUF7	Lj4g3v2400520	493	53.38	5.61	Lj4g	33042352..33042607	3	chlo	CBS domain-containing protein with a domain of unknown function (DUF21)
LjCBSDUF8	Lj5g3v2297970	488	53.17	6.04	Lj5g	33818491..33818668	3	plas	CBS domain-containing protein with a domain of unknown function (DUF21)
LjCBSDUF9	Lj6g3v1537040	302	34.12	5.64	Lj6g	17648484..17648546	0	vacu	CBS domain-containing protein with a domain of unknown function (DUF21)
LjCBSDUF10	Lj6g3v1886560	480	52.28	5.88	Lj6g	20896904..20897147	3	plas	CBS domain-containing protein with a domain of unknown function (DUF21)

**Fig. 1:**
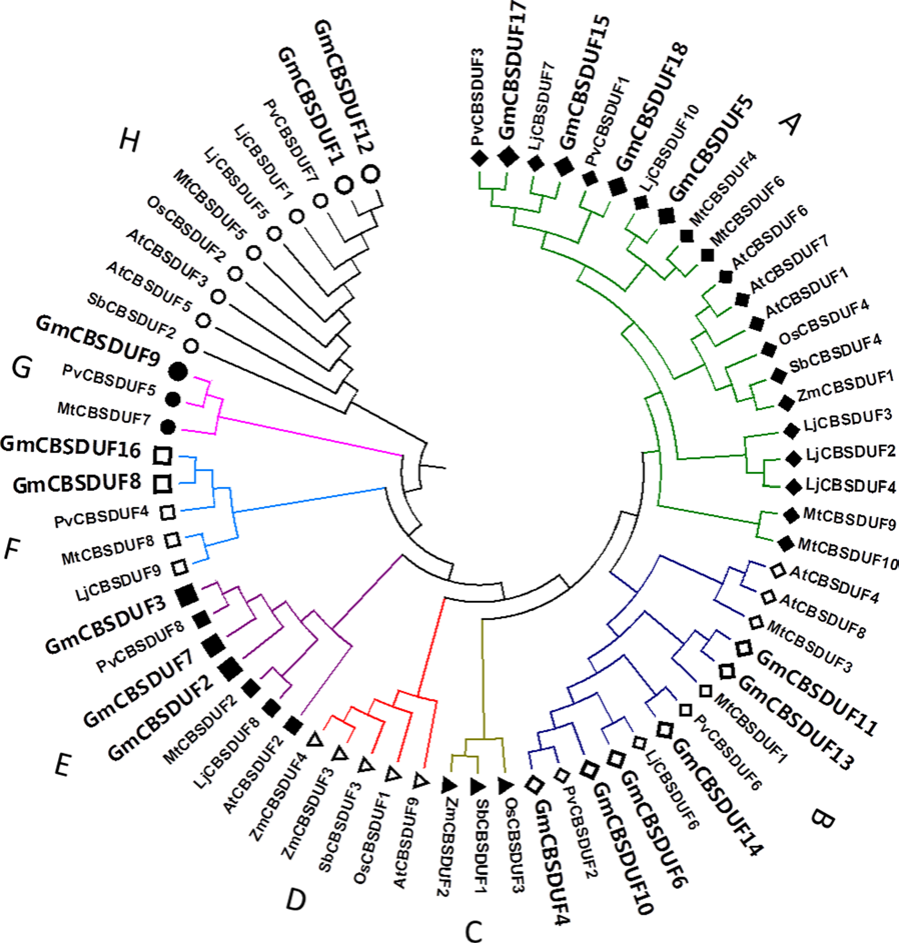
Phylogenetic relationships of the CBSDUFs. Phylogenetic relationships of the CBSDUFs from soybean (Gm), common bean (Pv), *Medicago truncatula* (Mt), *Lotus japonicus* (Lj), Arabidopsis (At), rice (Os), maize (Zm), and sorghum (Sb). The phylogenetic tree was constructed using Mega 6.0. The 67 CBSDUF proteins from eight plant species can be divided into eight groups (a–h); the branches are shown in different colors (Color figure online)

A phylogenetic tree was built with 67 protein sequences from eight plant species to investigate the phylogenetic relationships among CBSDUFs from soybean, three other legumes, Arabidopsis, and three gramineous plants (Fig. 1). The soybean CBSDUFs were named GmCBSDUF1 to GmCBSDUF18 according to their chromosomal positions. The genes from the other plant species were named by the same method. Based on the results of phylogenetic tree analysis, we divided these CBSDUFs into eight groups: Group A to Group H (Fig. 1). Group A included 21 members, and it covered eight species. All members of Group B and Group E were dicotyledonous plants. Group C was monocot-specific. Group D did not include legume members. Group F and Group G were legume-specific. The legume CBSDUFs show a very close evolutionary relationship, and the CBSDUFs from gramineous plants show a close evolutionary relationship. Compared to other species, the soybean CBSDUF gene family is extensively expanded. The number of soybean CBSDUFs was almost as many as those from rice, maize, sorghum, and Arabidopsis combined (Table 1). The number of GmCBSDUF genes is approximately two times more than those of Arabidopsis, common bean, *M. truncatula*, or *L. japonicus* and four times more abundant than those of rice, maize, or sorghum. The reason for this increase may be the multiple whole-genome duplication events of the soybean genome (Schmutz et al. 2010). The number of CBSDUF genes in dicotyledonous plants is much greater than that in monocotyledonous plants. Therefore, we speculate that CBSDUF plays an important role in dicots than monocots. The phylogenetic relationships may reflect some distinction between legume plant CBSDUFs and the four nonlegume plant CBSDUFs and indicate that the potential biological functions of some CBSDUFs are specific to legume plants.

### Gene Structure and Protein Structure of GmCBSDUFs

**Fig. 2:**
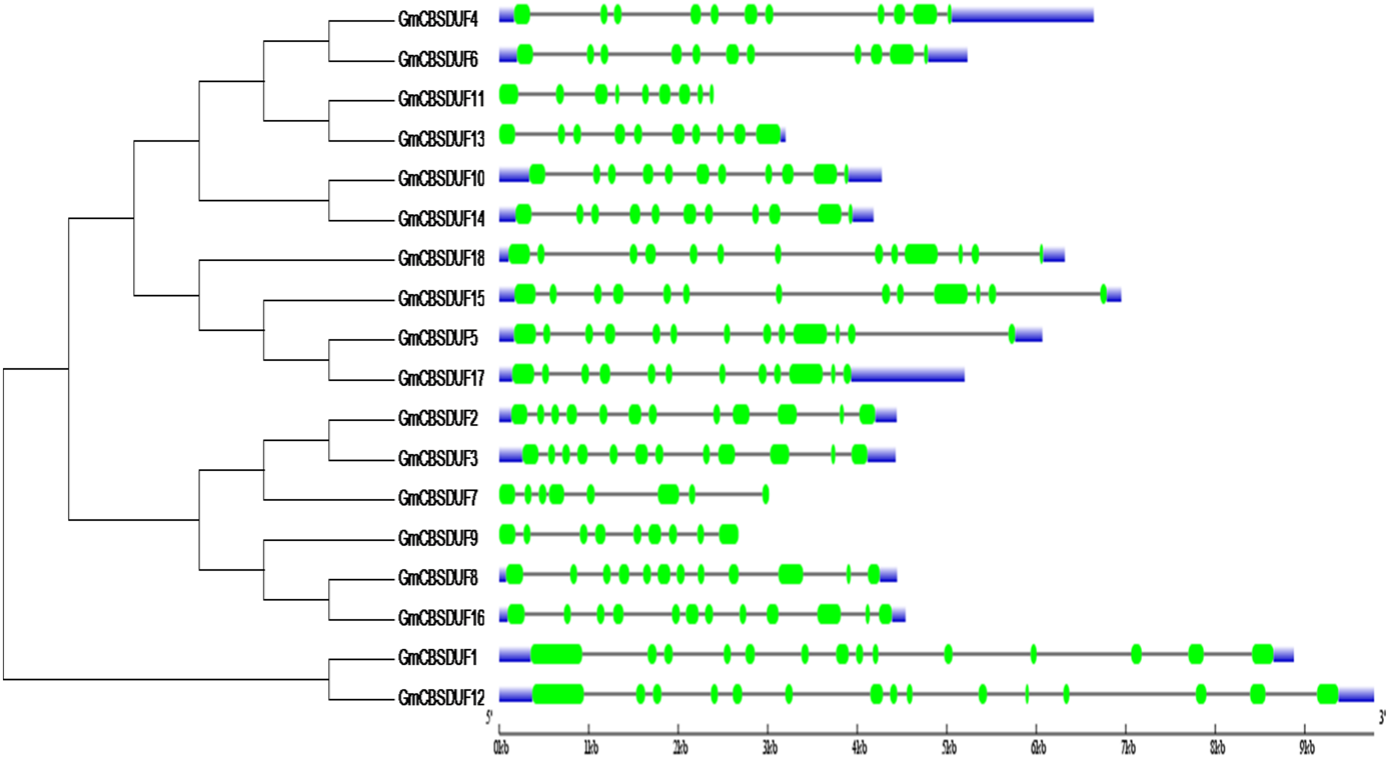
Phylogenetic relationships and gene structures of GmCBSDUFs. The phylogenetic tree (left panel) was constructed using MEGA 6.0, and the gene structures (right panel) were drawn using the gene structure display server

Exon–intron structural diversity often plays a key role in the evolution of gene families. To investigate the exon–intron organization of GmCBSDUFs, gene structures were mapped on the basis of the genomic and coding region sequences. The results showed that GmCBSDUFs have 8–15 exons and highly similar gene structures in the conserved region (Fig. 2). The size of GmCBSDUF genes is mainly affected by their intron size. GmCBSDUF12 is the largest gene and has the longest total intron length.

The soybean genome has undergone significant changes in the long-term evolutionary process. Some CBSDUF proteins are highly homologous in the terminal nodes, suggesting that they are putative paralogous pairs. In the study, a total of seven putative paralogous pairs (4/6, 10/14, 11/13, 5/17, 2/3, 8/16, 1/12) were identified, with sequence identities ranging from 60.47 to 99.26%.

**Fig. 3:**
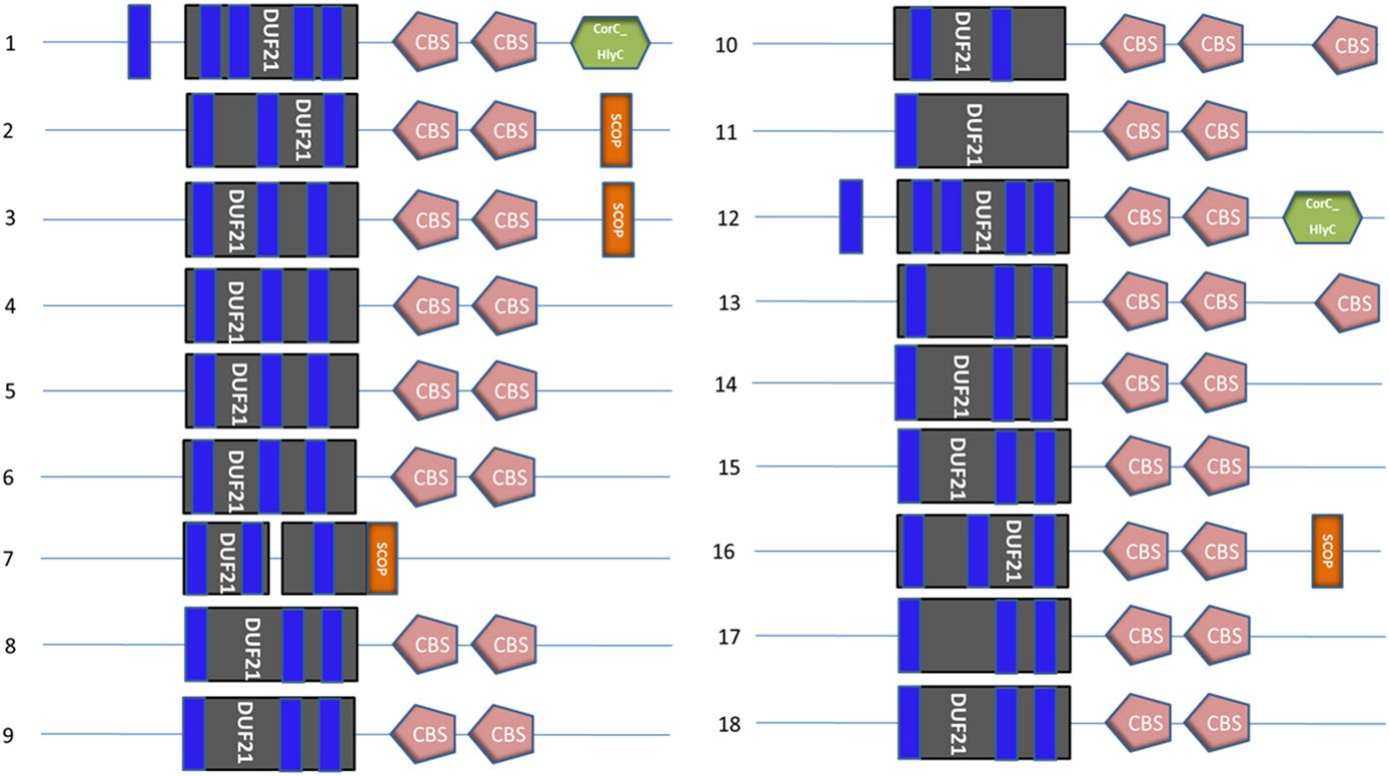
Main domains detected in soybean CBSDUF proteins by SMART. The blue rectangle represents the transmembrane region; the gray rectangle represents the DUF21 domain; the pink pentagon represents the CBS domain; the green hexagon represents the CorC_HlyC domain; and the orange rectangle represents the SCOP domain (Color figure online)

**Fig. 4:**
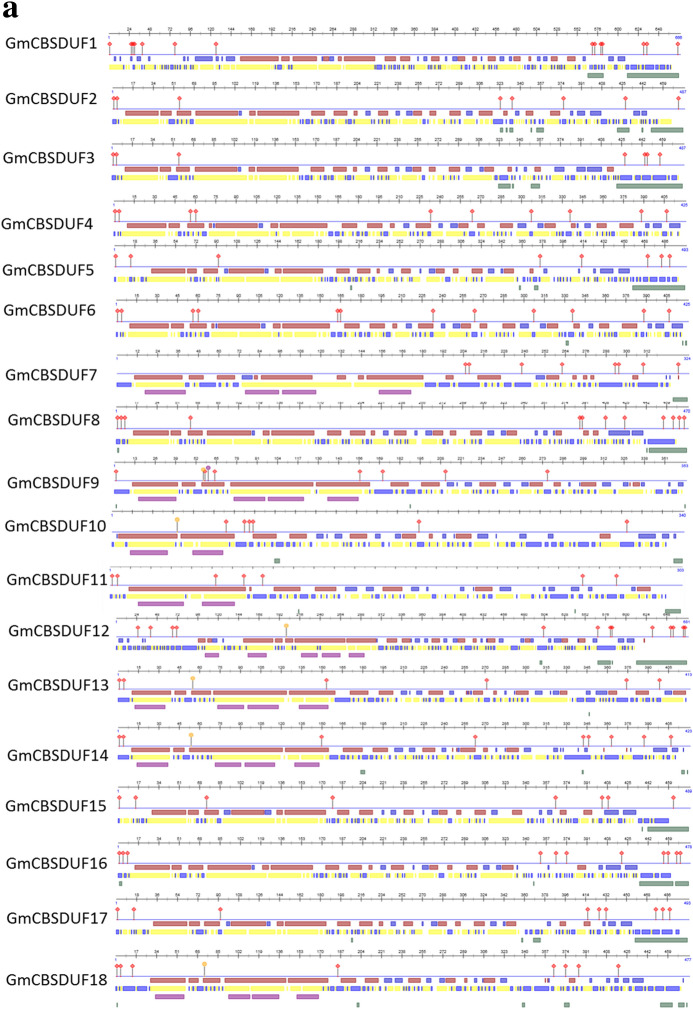

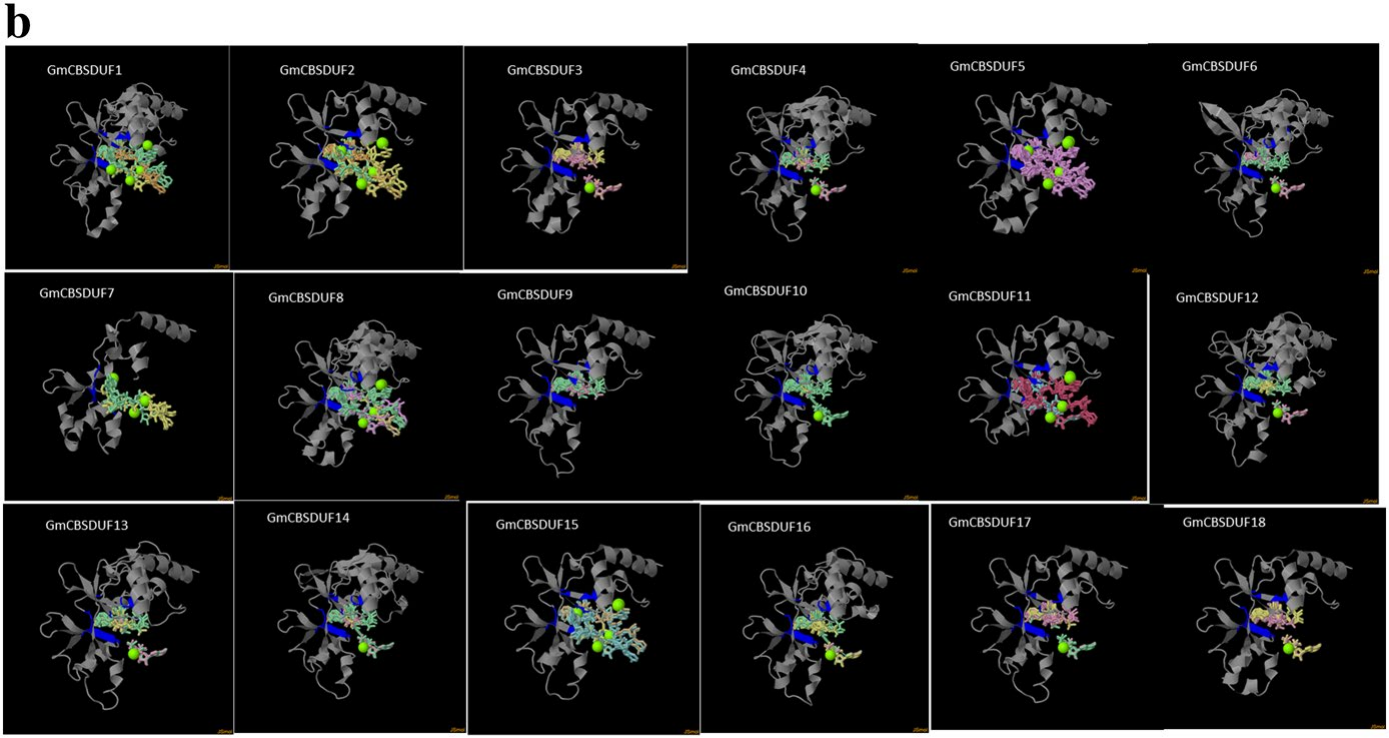
Protein structure analysis of soybean CBSDUF proteins. **a** The secondary structure analysis of soybean CBSDUF proteins.  protein binding region,  polynucelotide-binding region,  helix,  strand,  disordered region,  buried,  exposed,  helical transmembrane region. **b** The tertiary protein structures were predicted by using Phyre2 (Color figure online)

To some extent, functional information can be derived from structural similarity. Knowledge of the structure is often essential for interpreting functional data. GmCBSDUF protein structures are shown in Fig. S1. It is clear that GmCBSDUF proteins have a highly conserved hydrophobicity profile, with one hydrophobic segment located at the N terminus. SMART allows the identification and annotation of genetically mobile domains and the analysis of domain architectures. The results are shown in Fig. 3. The major domains are the DUF21 and CBS domains. The DUF21 domain is found in the N terminus of each protein, adjacent to two intracellular CBS domains, and has no known function. In addition, most GmCBSDUF proteins possess 3–4 transmembrane helices except for GmCBSDUF10, GmCBSDUF11, and GmCBSDUF13, which have 2, 1, and 5, respectively. Interestingly, all GmCBSDUFs transmembrane domains pass through the DUF21 domain. Therefore, we speculate that the domain of unknown function DUF21 may play a role in ion channel or signal transduction. In this study, the secondary and tertiary structures of GmCBSDUF proteins were predicted (Fig. 4). The structures were analyzed and compared to the results of Fig. 2. Proteins with high identities also have similar secondary structures, such as GmCBSDUF4/6, GmCBSDUF11/13, GmCBSDUF10/14, GmCBSDUF5/17, GmCBSDUF2/3, GmCBSDUF8/16, and GmCBSDUF1/12. Interaction with a ligand molecule is essential for many proteins to carry out their biological function. This interaction is generally specific, not only in terms of the molecules involved in the interaction but also in the location (i.e., the site of ligand binding) in which the interaction takes place. The results showed that although most GmCBSDUF proteins have similar structures, they have different binding sites, suggesting that they may display different functions.

### Tissue-Specific Expression Profiling of GmCBSDUFs

**Fig. 5:**
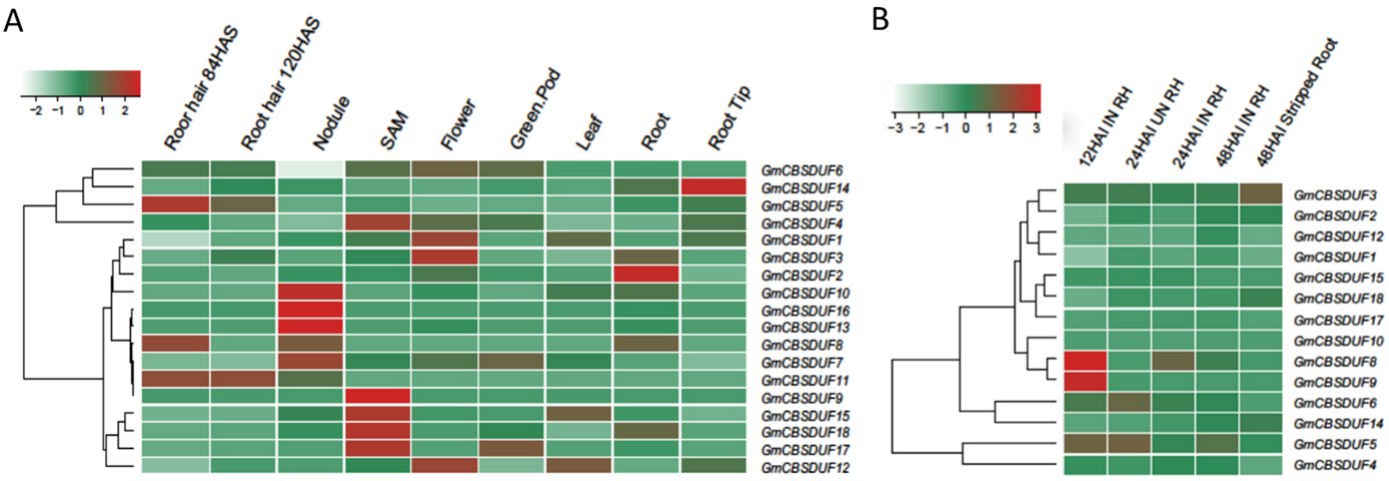
Tissue-specific expression profiles of GmCBSDUF genes. **a** Gene expression patterns of GmCBSDUF genes in nine different tissues, according to RNA-Seq data (Libault et al. 2010).* SAM* shoot apical meristem,* HAS* hours after sowing. **b** Comparison of the expression of soybean GmCBSDUF genes in root hairs (RH) and stripped roots inoculated (IN) and mock-inoculated (UN) with *B. japonicum* at 12, 24, and 48 h after *B. japonicum* inoculation (HAI). HAI IN RH: Root hair inoculated with *B. japonicum*; HAI UN RH: Root hair not inoculated by *B. japonicum*. Stripped roots: A soybean root after the stripping of root hairs. The color scale above the heat map indicates gene expression levels. The green color indicates a low expression level, and the red color indicates a high expression level (Color figure online)

Based on the publicly available soybean RNA-Seq data (Libault et al. 2010), the expression patterns of 18 GmCBSDUFs were investigated in various tissues, including (1) root hair cells isolated at 84 h after sowing (HAS), (2) root hair cells isolated at 120 HAS, (3) root tips, (4) roots, (5) mature nodules, (6) leaves, (7) shoot apical meristems, (8) flowers, and (9) green pods. An expression heat map was constructed (Fig. 5a). The results showed that (1) all GmCBSDUFs were expressed in at least one tissue; (2) GmCBSDUF2/3/5 were expressed in all tissues, and their expression levels were relatively high; (3) GmCBSDUF9 had the lowest expression under all conditions; (4) GmCBSDUF8 was expressed only in the underground tissues; and (5) GmCBSDUF9 was expressed only in one shoot apical meristem. In addition, GmCBSDUF1/12 as well as GmCBSDUF16/13 showed similar expression patterns. Moreover, based on the publicly available soybean RNA-Seq data (Libault et al. 2010), expression heat maps of 14 GmCBSDUFs (except GmCBSDUF7/11/13/16, which were not or barely expressed in roots) in root hairs harvested at 12, 24, and 48 h after *Bradyrhizobium japonicum* inoculation (HAI), in mock-inoculated root hairs at 24 HAI, and in stripped roots at 48 HAI were also constructed (Fig. 5b). Based on the rhizobial inoculation method according to Libault et al. (2010), a *B. japonicum* cell suspension or water (mock inoculation) was sprayed on soybean seedlings growing on B&D agar medium. The results showed that inoculation with *B. japonicum* significantly increased the expression of GmCBSDUF8/9, but not other GmCBSDUFs, in root hairs. Therefore, we suspect that GmCBSDUF8/9 may be required for bacterial recognition, nodulation, and nitrogen fixation.

Furthermore, the soybean (*Glycine max*) genome database (Phytozome 12) provides high-resolution gene expression data for a diverse set of 17 soybean GeneAtlas tissue samples, such as flower (open and unopened), lateral root (standard), leaf (ammonia, nitrate, urea, standard and symbiotic condition), nodule (symbiotic condition), root tip (standard), root (ammonia, nitrate, urea, standard and symbiotic condition), shoot tip (standard), stem (standard), and 9 soybean normal tissue samples (flower, leaf, nodule, pod, root, root hair, seed, SAM, and stem). These data were also analyzed and represented as heat maps (Fig. S3). Expression analyses of all GmCBSDUF genes revealed that the different members have different tissue-specific expression. Among all 18 analyzed genes, GmCBSDUF5 showed the highest level of constitutive expression in all tissues, followed by GmCBSDUF3, GmCBSDUF2, and GmCBSDUF12. This high level of constitutive expression indicates a significant role in all these soybean tissues (Fig. S3). A cluster of genes showed low levels of expression in all tissues. They are GmCBSDUF8/9/11/13. GmCBSDUF16 is highly expressed only in root nodules, but its expression is very low in symbiotic conditions. These results are basically consistent with the results in Fig. 5, which makes the analysis of tissue expression patterns of GmCBSDUF genes more sufficient and meaningful. Analysis of the expression patterns of these genes will be helpful to the study of their function. All these expression profiles suggest functional redundancy and divergence among the soybean GmCBSDUFs during plant growth and development.

### Promoter Analysis

Based on the soybean genome database (https://www.phytozome.net/soybean), the promoter regions located 2 kb upstream of the translation start codons of the GmCBSDUF genes were analyzed using the PlantCARE promoter analysis program (https://bioinformatics.psb.ugent.be/webtools/plantcare/html/). Multiple elements were identified, and the stress and hormone signaling-related sites are shown in Table 2. The table describes information pertaining to functions, such as elements in response to hormones, including abscisic acid (ABRE, CE1, and MRE) (Narusaka et al. 2003), salicylic acid (TCA element) (Liu et al. 2020), ethylene (ERE) (Song et al. 2019), gibberellin acid (GARE-motif, P-box and TATC-box) (Zhang et al. 2017), auxin (TGA-element) (Xin et al. 2016), MeJA (CGTCA-motif and TGACG-motif) (Yu et al. 2018), temperature-responsive elements (HSE and LTR) (Wu et al . 2019), drought-inducible elements (MBS) (Xu et al. 2019), wound-responsive element (WUN-motif), defense and stress element (TC-rich repeats) (Li et al. 2019), salt-inducible element (GT1-motif), anaerobic induction element (ARE), and light- and nitrogen-inducible element (GATA-motif) (Brenna and Talora 2019). As shown in Table 2, ABRE, MBS, TCA element, GARE-motif, and HSE were all present in the promoters of most of the GmCBSDUF genes, while the WUN-motif was found only in GmCBSDUF2; P-box in GmCBSDUF10; CE1 in GmCBSDUF16; TATC-box in GmCBSDUF4/6; LTR in GmCBSDUF9/13/18; and GATA-motif in GmCBSDUF5/6/10/13/15/17. The prediction of promoter elements provided some clues to the responses of GmCBSDUFs to various abiotic stresses.

**Table 2:** Stress-related potential cis element sites found in GmCBSDUFs promoters

Elements	Sequences	Functions	GmCBSDUF1	GmCBSDUF2	GmCBSDUF3	GmCBSDUF4	GmCBSDUF5	GmCBSDUF6	GmCBSDUF7	GmCBSDUF8
ABRE	CACGTG	ABA, dehydration	2	1	1	2	2	1	1	2
4MBS	CAACTG	Drought	3	1	2	0	0	1	5	1
TCA element	GAGAAGAATA	Salicylic acid	4	3	0	2	1	1	1	0
ERE	ATTTCAAA	Ethylene	2	1	0	0	0	0	0	0
GARE-motif	AAACAGA	Gibberellin	1	1	2	0	2	0	4	1
TC-rich repeats	ATTTTCTTCA	Defense and stress	0	1	3	1	3	2	1	2
WUN-motif	TCATTACGAA	Wounding	0	1	0	0	0	0	0	0
CGTCA-motif	CGTCA	MeJA	0	0	1	1	0	1	1	1
GT1-motif	GGTTAA	Disease, salt	0	0	3	1	1	2	0	0
TGACG-motif	TGACG	MeJA	0	0	1	1	0	1	1	1
ARE	TGGTTT	Anaerobic induction	0	1	1	4	1	1	2	1
TATC-box	TATCCCA	Gibberellin	0	0	0	1	0	1	0	0
LTR	CCGAAA	Low temperature	0	0	0	0	0	0	0	0
GATA-motif	GATAGGA	Light, nitrogen	0	0	0	0	1	1	0	0
P-box	CCTTTTG	Gibberellin	0	0	0	0	0	0	0	0
TGA-element	AACGAC	Auxin	0	0	0	0	1	0	0	0
MRE	AACCTAA	ABA, stress	0	0	0	0	0	0	0	0
CE1	TGCCACCGG	ABA	0	0	0	0	0	0	0	0
HSE	AAAAAATTTC	Heat	4	0	5	1	4	2	1	2

Elements	GmCBSDUF9	GmCBSDUF10	GmCBSDUF11	GmCBSDUF12	GmCBSDUF13	GmCBSDUF14	GmCBSDUF15	GmCBSDUF16	GmCBSDUF17	GmCBSDUF18
ABRE	3	0	0	0	1	0	0	2	1	0
4MBS	1	0	2	0	1	0	0	1	3	2
TCA element	1	4	2	1	1	3	1	2	0	0
ERE	1	1	0	2	0	1	0	0	1	0
GARE-motif	0	1	1	2	1	0	1	0	3	1
TC-rich repeats	2	2	0	1	0	2	1	0	1	0
WUN-motif	0	0	0	0	0	0	0	0	0	0
CGTCA-motif	1	0	0	2	0	0	1	1	2	1
GT1-motif	3	2	2	1	3	0	3	0	0	3
TGACG-motif	1	0	0	1	0	0	1	1	2	0
ARE	0	0	1	1	1	0	4	0	2	1
TATC-box	0	0	0	0	0	0	0	0	0	0
LTR	1	0	0	0	1	0	0	0	0	1
GATA-motif	0	2	0	0	1	0	2	0	1	0
P-box	0	2	0	0	0	0	0	0	0	0
TGA-element	0	0	1	1	0	1	0	0	0	1
MRE	0	0	0	0	0	1	1	0	0	0
CE1	0	0	0	0	0	0	0	1	0	0
HSE	4	5	1	6	1	1	2	1	3	2

### Expression Profiles of GmCBSDUFs Under Low Nitrogen Stress Conditions

**Fig. 6:**
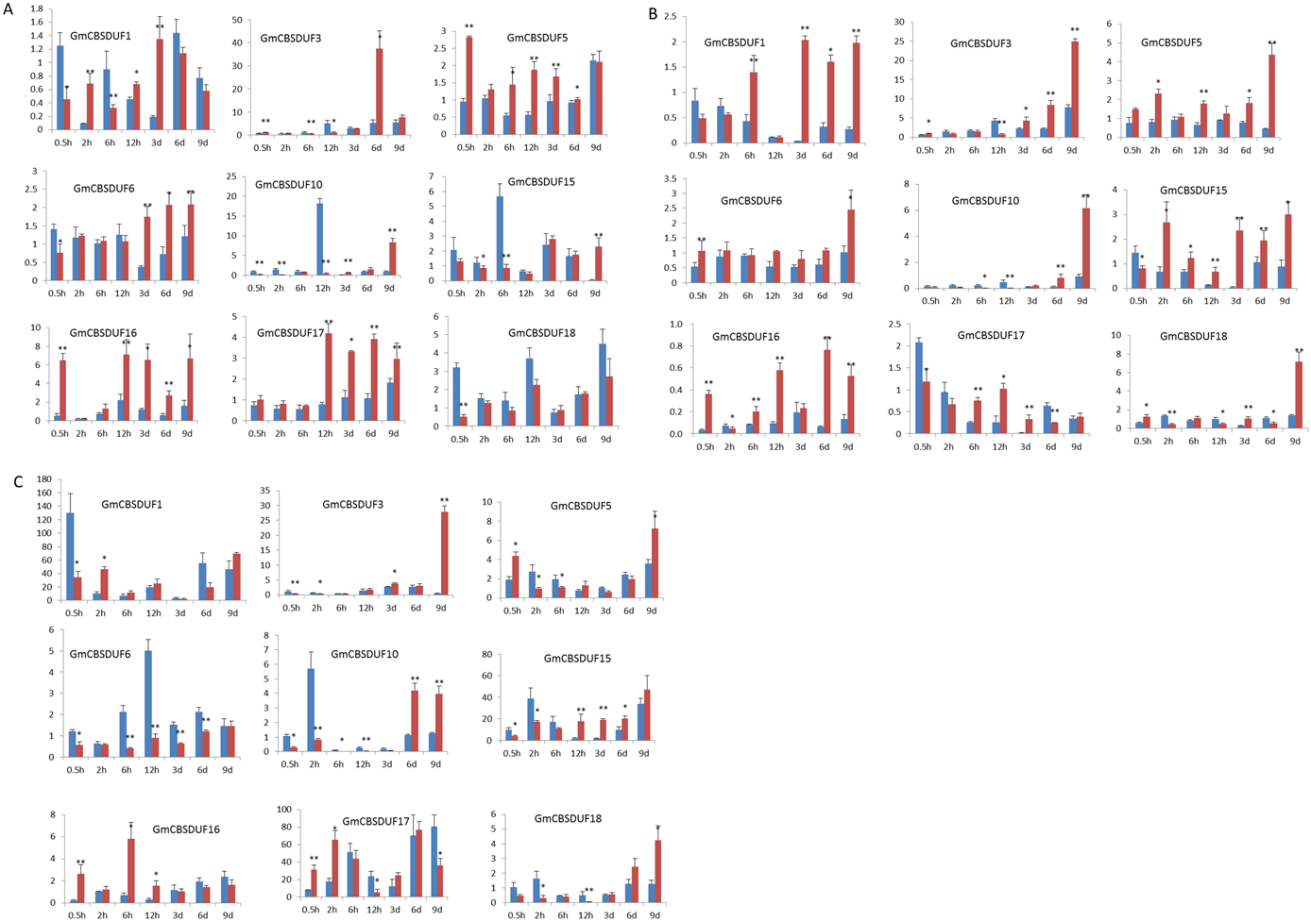
Expression of nine soybean GmCBSDUF genes in response to low nitrogen stresses. **a** Leaves, **b** stems, **c** roots. Data were obtained by real-time PCR normalized against the reference gene ACT11 and are shown as a percentage of expression in the control at 0 h. Blue columns represent the expression under normal nitrogen conditions, and red columns represent the expression under low nitrogen conditions. GmCBSDUF13, which was not expressed in soybean roots, stems, and leaves under normal conditions, was not induced under low nitrogen stress and was not present in this figure (Color figure online)

Our previous studies have shown that GmCBS21, which contains the DUF21 and CBS domains, can improve plant low nitrogen tolerance (Hao et al. 2016). To further understand the low nitrogen responses of GmCBSDUF genes, the transcript levels of these genes in soybean seedlings under low and normal nitrogen conditions were analyzed using real-time PCR. Figure 6a–c shows their expression in leaves, stems, and roots, respectively, at 0.5 h, 2 h, 6 h, and 12 h (short-term) and 3, 6, and 9 days (long-term) post-treatment.

As shown in Figs. 6a and S4, 17 soybean GmCBSDUF genes were differentially expressed in the leaves of low-nitrogen-treated seedlings and untreated control seedlings. The expression patterns of the soybean GmCBSDUF genes in leaves were very different from those in stems and roots. As shown in Fig. 6a, (1) GmCBSDUF16 and GmCBSDUF17 were upregulated after low nitrogen treatment at all time points, (2) GmCBSDUF18 was downregulated after low nitrogen treatment at most of the time points, only 3 and 6 days were slightly increased (3) GmCBSDUF5 was upregulated after 0.5 h to 6 days of low nitrogen treatment but slightly downregulated after 9 days of low nitrogen treatment, and (4) GmCBSDUF10/15 was downregulated after short-term treatment but upregulated after long-term treatment. These results may indicate that these genes play different roles in different time periods.

Figures 6b and S4 show the expression of GmCBSDUF genes in stems. It was clear that 17 GmCBSDUF genes were differentially expressed in stems after low nitrogen treatment. Among them, the expression of GmCBSDUF5 and GmCBSDUF11 was significantly upregulated at low nitrogen conditions at any given time point; the expression of GmCBSDUF6 and GmCBSDUF9 was significantly upregulated at most time points, and the difference was not significant only at the 6 h point. The expression of GmCBSDUF15 was significantly upregulated at 6 time points except at 0.5 h (downregulated). The expression levels of GmCBSDUF4 and GmCBSDUF12 were upregulated at most time points except 12 h (downregulated). The expression levels of 4 GmCBSDUF genes (GmCBSDUF5/7/8/11) were upregulated after short-term treatment, and 7 GmCBSDUF genes (GmCBSDUF1/3/4/5/12/15/16) were upregulated after long-term treatment.

Figures 6c and S4 show the expression of GmCBSDUF genes in roots. In detail, low nitrogen conditions significantly upregulated the expression of GmCBSDUF2/8/11 but downregulated the expression of GmCBSDUF4/6/7/14. Moreover, GmCBSDUF3/10/15/18 increased after long-term treatment (6, 9 days) while expression of GmCBSDUF16 decreased.

The above results clearly showed that most GmCBSDUF genes were significantly induced in response to low nitrogen stress treatment. Therefore, we speculate that, in addition to the GmCBS21 gene, the other genes in the family are also associated with plant nitrogen utilization. We also found significant gene expression changes in leaves at the early time point (0.5 h) after stress treatment. This may indicate that these genes play a major role in nitrogen assimilation. Future studies are needed to demonstrate the functional roles of genes responsive to low N stress in relation to N metabolism.

### Effect of Abiotic Stresses on the Expression of GmCBSDUFs

As described in Table 2, most soybean GmCBSDUF genes have stress and hormone signaling-related responsive elements. Some studies have also found a role for plant CDCPs in abiotic stress response (Kushwaha et al. 2009). To investigate whether GmCBSDUFs also have similar roles in soybean, the expression patterns of GmCBSDUFs in response to cold, dehydration, H_2_O_2_, ABA, and salinity stress were examined. The raw expression values for the genes are shown in Table S2.

**Fig. 7:**
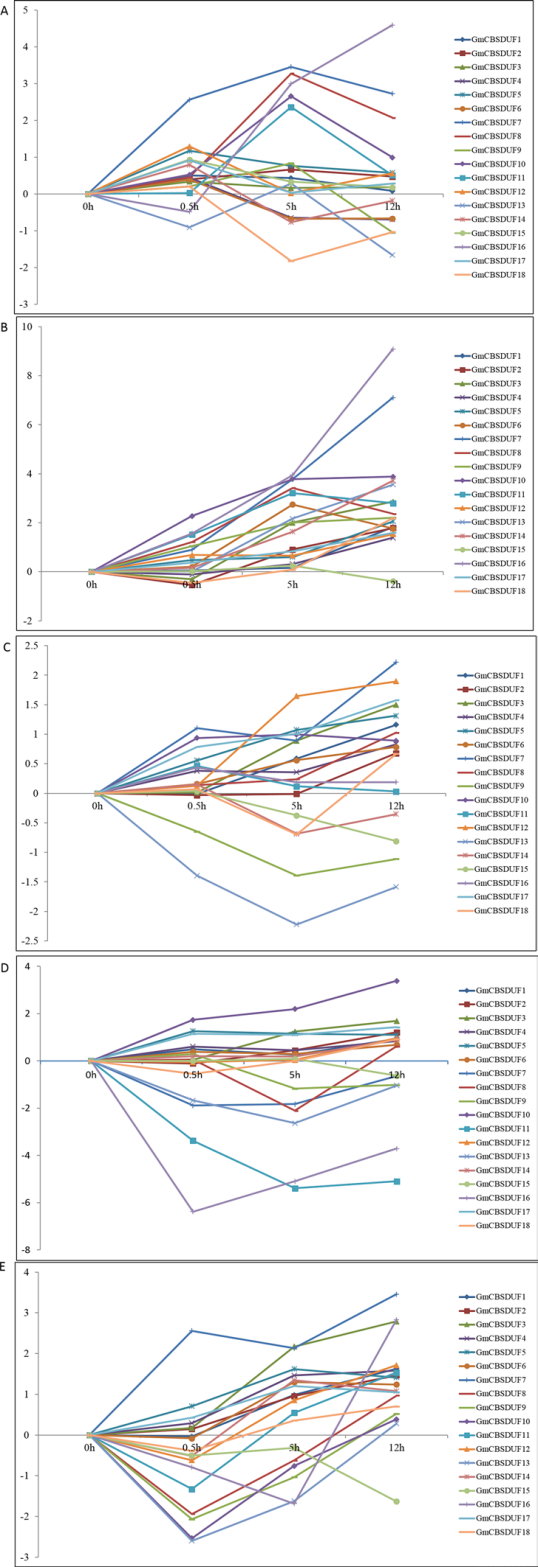
Expression analysis of GmCBSDUF genes in response to abiotic stresses. Two-week-old soybean seedlings were exposed to stress treatments as indicated below. Gene expression analysis was conducted by qRT-PCR using gene-specific primers. **a** Cold stress, **b** dehydration stress, **c** H_2_O_2_ stress, **d** ABA stress, **e** salinity stress. The transcript levels of GmCBSDUF genes in plants at 0.5, 5, and 12 h poststress treatments were plotted as the relative expression (fold change) of the nonstressed control plants. The transcript level of actin was used as a reference

Two-week-old soybean seedlings were exposed to cold stress at 4 °C for 0, 0.5, 5, or 12 h, and the expression of GmCBSDUFs was detected. The results revealed that cold stress altered the expression of GmCBSDUFs, which could be grouped into 3 categories. As indicated in Fig. 7a, category I contained genes that showed increased transcript accumulation under stress, including GmCBSDUF7/8/11/13/16, and the expression of GmCBSDUF7/8/11 decreased slightly at 12 h. All four gene family members were expressed to their highest level either at 5 or 12 h after cold stress. Category II contained genes (GmCBSDUF2/3/4/5/6/17/18) that showed a gradual decrease in transcript accumulation with prolonged cold treatment. In addition, the expression of GmCBSDUF10 reached its lowest level at 0.5 h, and GmCBSDUF12 reached its highest at level 0.5 h. The expression levels of genes in category III (GmCBSDUF 1/9/14/15) showed no obvious change.

Figure 7b shows the effects of dehydration treatment on the transcription of GmCBSDUFs in soybean seedlings. It is clear that (1) the transcript levels of 18 GmCBSDUFs gradually increased with prolonged stress. Among the 18 GmCBSDUF genes, GmCBSDUF1/2/3/4/5/12/18 were only weakly upregulated (no more than threefold) under dehydration treatment. GmCBSDUF6/8/9 peaked at 5 h, and 2/5/10/17 decreased at 0.5 h. By comparison, GmCBSDUF7/8/9/10/11/13/14/16 showed notable changes. (2) The transcript levels of GmCBSDUF15 and GmCBSDUF17 were slightly downregulated under dehydration treatment. These results further suggest that GmCBSDUF genes play a role in plant drought resistance.

Figure 7c shows the effects of H_2_O_2_ on the transcription of GmCBSDUFs in the roots of soybean seedlings. It is clear that H_2_O_2_ treatment (1) increased the transcript levels of GmCBSDUF6/7/8/10/11/12/16, (2) decreased the transcript levels of GmCBSDUF2/3/14/15/17/18, and (3) did not change the transcript levels of other GmCBSDUFs.

Figure 7d shows the time-course effects of 100 μM ABA on the transcription of GmCBSDUFs in soybean seedlings. The results show that (1) the expression levels of GmCBSDUF9 and GmCBSDUF11 were significantly increased by 100 μM ABA treatment at 0.5 h but gradually decreased with prolonged ABA, (2) the expression levels of GmCBSDUF2/4/10 were significantly increased with ABA treatment, and (3) the expression levels of GmCBSDUF15/17/18 were significantly decreased after ABA treatment.

Figure 7e shows the time-course effects of salt stress on the transcription of GmCBSDUFs in soybean seedlings. The results show that (1) the expression levels of GmCBSDUF 1/2/3/4/8/9/10 gradually increased as the stress was prolonged, and GmCBSDUF8/9/10 reached their highest levels at 12 h of salt stress, while GmCBSDUF1/2/3/4 reached their lowest levels at 0.5 h of salt stress; (2) the expression levels of GmCBSDUF7/11/13/14 increased considerably at one or more stress time points (0.5 h, 5 h, or 12 h), and (3) the expression levels of GmCBSDUF15/17/18 decreased compared to the 0 h treatment.

### Phenotypes of GmCBSDUF3 Transgenic Arabidopsis

**Fig. 8:**
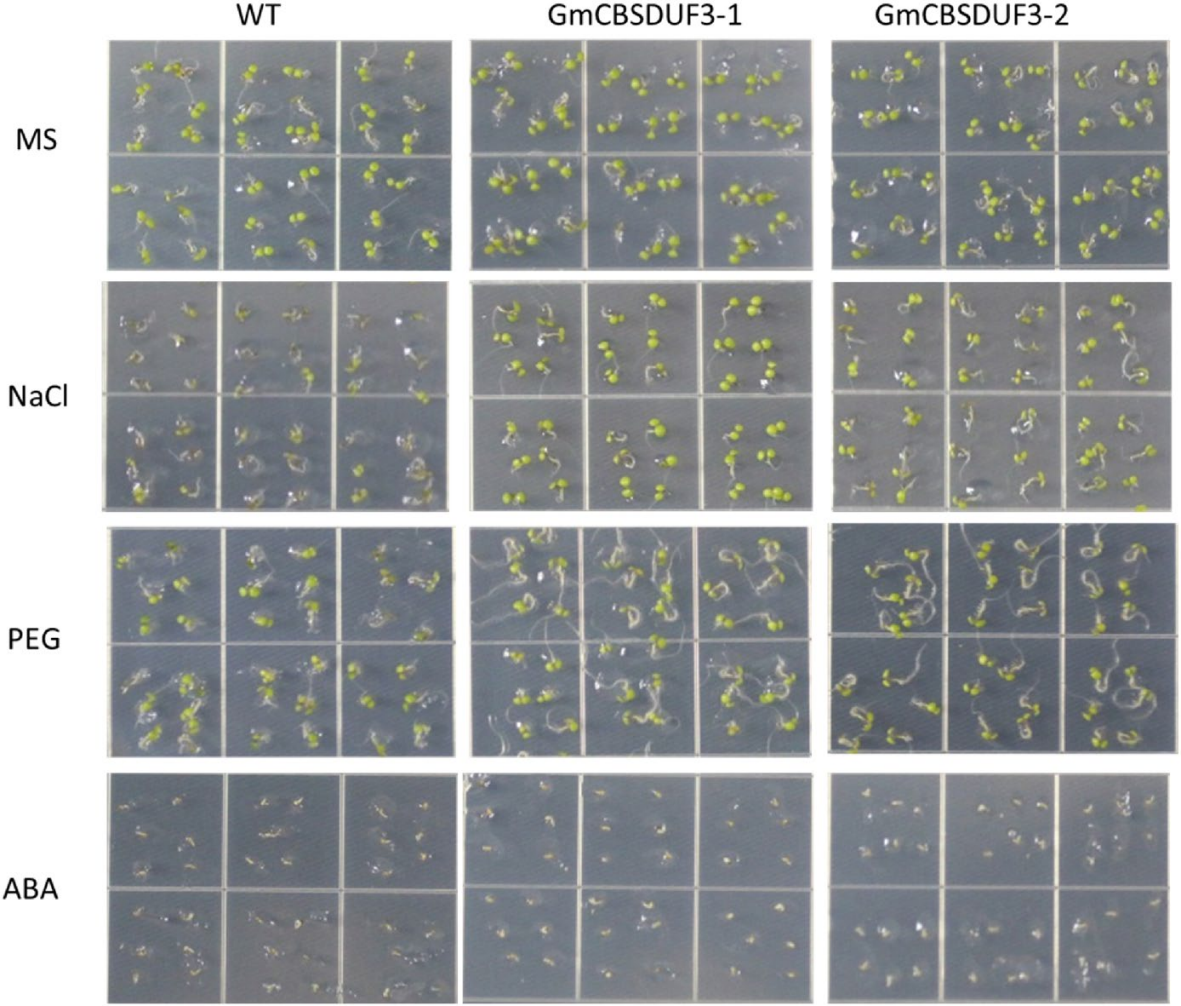
Effect of salt, drought and ABA stresses on seed germination of GmCBSDUF3 transgenic and WT seeds

**Fig. 9:**
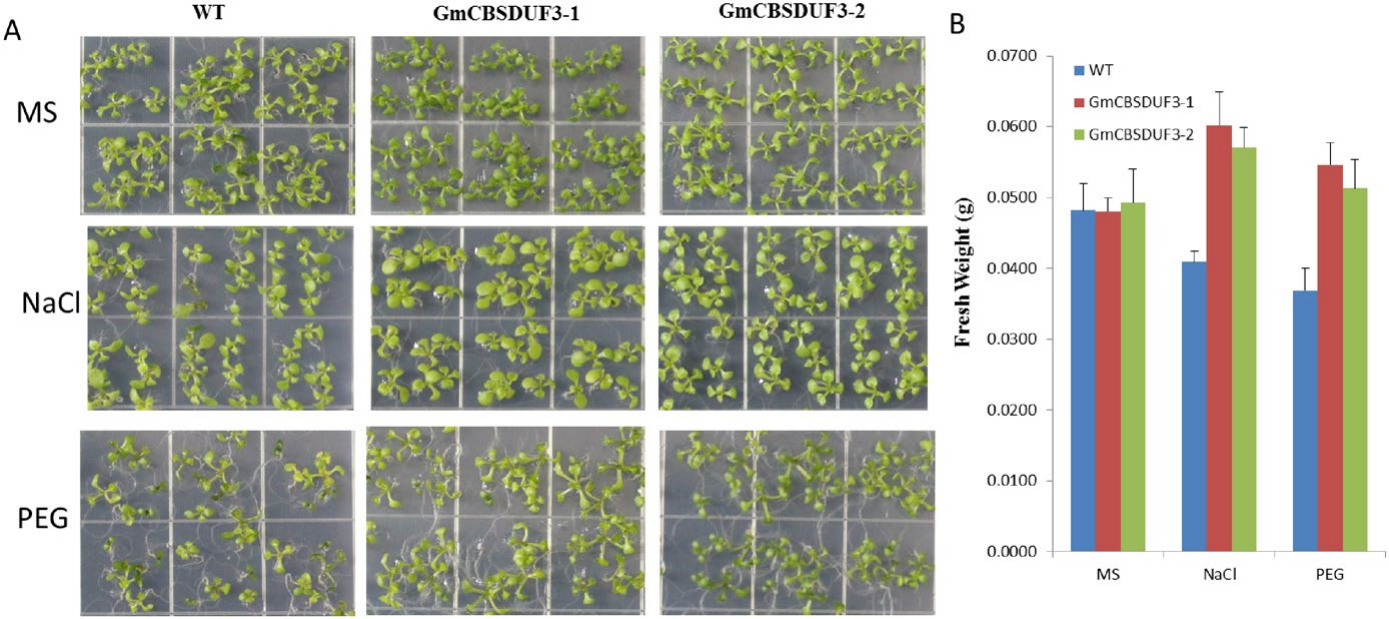
Effect of salt and drought stresses on GmCBSDUF3 transgenic and WT seedlings. **a** The phenotypes of GmCBSDUF3 transgenic and WT seedlings under salt and drought stresses. **b** Statistical analysis of fresh weights

CBSDUFs may be involved in multiple stress responses in plants. As described above, when induced with some stresses, the expression of GmCBSDUF genes is significantly altered. Our previous study found that GmCBSDUF3 could improve plant nitrogen use efficiency. Therefore, we chose GmCBSDUF3 for further functional exploration. Two homozygous constitutively overexpressing Arabidopsis lines (GmCBSDUF3-1 and GmCBSDUF3-2) with higher GmCBSDUF3 expression were selected for phenotypic analysis under NaCl, PEG, and ABA stress treatments. As shown in Fig. 8, on MS medium alone, no obvious difference was observed between the transgenic and wild-type (WT) seeds. However, when sown on MS medium containing 50 mM NaCl, WT seeds germinated much later than transgenic GmCBSDUF3 seeds. After sowing on MS medium containing 2% PEG for 5 days, transgenic plants grew better than WT plants and had well-developed root systems. The germination rate on MS medium containing 1.5 μM ABA was also analyzed. Treatment with ABA delayed the germination of both transgenic and WT seeds and led to no significant difference between the transgenic and WT plants. The transgenic plants and control plants were also sensitive to ABA stress. After 10 days of treatment, the growth status of GmCBSDUF3 transgenic Arabidopsis seedlings was also investigated. As shown in Fig. 9a, when the seedlings were grown on MS medium supplemented with 50 mM NaCl or 2% PEG, transgenic plant growth was superior to that of WT. Transgenic plants had well-developed root systems to absorb nutrients and water. Groups of ten seedlings per strain were used to measure the whole plant weight (fresh weight). The fresh weights of the transgenic seedlings were higher than those of WT (Fig. 9b). Because of the well-developed root system under NaCl or PEG conditions, the transgenic seedling weight is higher than under normal conditions. These results revealed that overexpressing GmCBSDUF3 in plants could increase tolerance under NaCl and PEG stress conditions.

## Discussion

Although some CDCPs, such as IMPDH (Collart et al. 1996; Wang et al. 2004) and ClC (Hechenberger et al. 1996; Diédhiou and Golldack 2006; Lv et al. 2009), have been characterized in plant systems, the majority of members in this family remain uninvestigated, especially the CBSDUF subgroup. Many sequences related to CBSDUF genes have been uploaded in GenBank, but only a few of them have been well described in terms of their expression pattern, biochemical characteristics, subcellular locations, and particularly their biological functions. Transcriptomic and proteomic analyses of CDCPs have revealed differential expressional profiles in plants challenged with virus (Espinoza et al. 2007), fungi (Fabro et al. 2008), salinity stress (Kumari et al. 2009; Sahu and Shaw 2009), and oxalic acid treatment (Wang et al. 2009). All these data indicate that the members of this family in different plant species may play important roles in diverse developmental processes, including developmental programmed cell death, and responses to different biotic and abiotic stresses. These works present the necessity of extensively investigating CBSDUF genes in plants, especially in crops, with the expectation of improving crop yield and resistance. They have identified, classified, and suggested the nomenclature of CDCPs in Arabidopsis and rice and performed a brief analysis of expression patterns for CDCPs using the already existing transcriptome profiles and the MPSS database (Kushwaha et al. 2009). However, the detailed expression characteristics of CBSDUF subgroup genes in plants, especially in soybean, are still largely unknown. In this study, 18 CBSDUF genes were identified in the soybean genome through the public genome database. The characteristics of CBSDUF genes were analyzed in detail in our study.

### Characteristics of CBSDUF Genes in Soybean

Bioinformatics analysis has become the first and most important method for the study of new gene functions. By bioinformatics analysis, researchers can often obtain important information about the functions of new genes and then make a plan for further experimental research. Therefore, we analyzed the structures and molecular evolution of GmCBSDUF genes as well as their coding products and structures. The relatively higher number of CBSDUF-family genes in soybean is consistent with the suggestion that gene duplication has been universal in the soybean genome during its evolution (Schmutz et al. 2010). By domain analysis, we found that a highly conserved DUF21 domain exists only with the CBS domain. This domain may be crucial for GmCBSDUF gene function. To carry out research on the functions of new genes, we must first clarify their regular gene expression patterns in vivo. Thus, the expression patterns of GmCBSDUF genes were analyzed in different developmental stages and tissues of soybean (Fig. 5). The results revealed the tissue-specific expression patterns of CBSDUF genes in soybean. Some GmCBSDUF genes were maintained at high expression levels in some plant tissues, followed by moderate expression levels in other tissues (Fig. 5a). For example, GmCBSDUF14 was highly expressed in the root tip, while GmCBSDUF2 was highly expressed in the root. In contrast, some GmCBSDUF genes, such as GmCBSDUF8, GmCBSDUF11, and GmCBSDUF16, showed low expression levels in only the underground tissues with no expression in other tissues. This implies that different GmCBSDUF genes may have different functions in different tissues. A *M. truncatula* CBSDUF protein, MtCBS1, was found to be required for rhizobial infection and symbiotic nitrogen fixation (Sinharoy and Liu 2016). GmCBSDUF8 is the closest homolog of MtCBS1 in soybean and is expressed in only roots. After inoculation of *B. japonicum*, its expression was induced in root hairs, suggesting a potential role of GmCBSDUF8 in symbiosomes capable of fixing nitrogen. We will further verify this function by experiment.

### Potential Roles of CBSDUF Genes in Response to Different Stress Treatments

It is well known that plant responses and stress-activated signaling pathways are largely overlapping. Kushwaha et al. (2009) reported that some AtCBS genes, such as AtCBSX2, AtCBSX3, and AtCBSCBS1, were stably expressed under any stress conditions, while some, such as AtCBSX1 and 15, were more sensitive to all stress conditions in both roots and shoots, and some, such as AtCBSDUFCH2, AtCBSDUF1, AtCBSDUF2, and AtCBSCBS2, were sensitive to stress conditions only in roots. In this study, the expression patterns of soybean CBSDUF genes under abiotic stresses were analyzed (Fig. 6). In contrast to other subgroup members, the results showed that GmCBSDUF7/8/11/16 was upregulated after exposure to cold, drought, salt, and H_2_O_2_, while GmCBSDUF17/18 was downregulated by cold, H_2_O_2_, salt and ABA, suggesting that these GmCBSDUF genes may play a role in crosstalk between signaling pathways responding to drought, H_2_O_2_, salinity, cold, and ABA. The results presented here will be helpful for future studies of the biological functions of GmCBSDUF proteins. Remarkably, we found that GmCBSDUF7/8/11/13/16 showed significant differences in expression under stress treatments. Therefore, we speculate that these genes are inducible and may play an important role in stress response. We will further examine this prospect in subsequent studies.

In conclusion, we performed a comprehensive bioinformatics analysis and provided detailed information on the soybean CBSDUF gene subgroup. Specifically, our results show that the soybean genome contains 18 CBSDUF genes, the largest subgroup among the identified CBSDUF gene subgroups in the study. Our analysis revealed the possible function of each GmCBSDUF gene in response to cold, salt, H_2_O_2_, ABA, dehydration, and low nitrogen, identified their potential clients and functional interactions, and revealed the specific responses of some GmCBSDUF genes to specific stresses. By interaction network prediction, some candidate interacting genes were found. At the same time, we preliminarily explored the function of GmCBSDUF3, which might improve the ability to resist abiotic stress in plants. This result provides an impetus for additional investigation of the biological roles and interacting proteins of the CBSDUF protein family in soybean, and a functional analysis of the genes in this family will be carried out systematically. In the future, we will use functional genomics in combination with a transgenic approach to verify the utility of those proteins with defined features as tools to improve stress tolerance in crop plants. Based on the present research and the characteristics of each family member, the research on functional analysis was classified and summarized. We will use gene knockout and transgenic technology to study the functions of the GmCBSDUFs. At the same time, the functions of the two domains, CBS and DUF21, will be studied by site-directed mutagenesis. In addition, due to the lack of information about this family of proteins, the biological pathways involving these genes are still unknown. We will screen for interacting proteins with yeast two-hybrid technology and provide evidence for their mechanisms of action. We will also determine the expression of transgenic plants under specific conditions by high-throughput sequencing technology and infer the gene regulatory network. The ideas provided here would also have a way for expounding the definite role of CBSDUF proteins in plants.

## Materials and Methods

### Identification of DUF21 and CBS Domain-Containing Proteins in Soybean

The known DUF21 and CBS domain-containing protein sequences from soybean, Arabidopsis, common bean, *M. truncatula*, *L. japonicus*, rice, maize, and sorghum were obtained from the NCBI database and used as queries to conduct BLAST searches against the public genome database (https://phytozome.jgi.doe.gov/pz/portal.html#) and *L. japonicus* genome database (https://www.kazusa.or.jp/lotus/). Sequences with an *E* value < 1.0 were selected for further analysis. A search with the keywords PF00571 for the CBS domain and PF01595 for the DUF21 domain was conducted for putative soybean CBSDUFs by searching ontologies against the Phytozome (v12.0) database (https://www.phytozome.net). If more than one transcript existed, the primary transcript was selected as a representative.

### Phylogenetic, Gene, and Protein Structure Analyses

Multiple alignment analysis was performed with ClustalX 1.83 software (Thompson et al. 1997). Phylogenetic trees were generated by the neighbor-joining (NJ) method and bootstrap analysis (1000 replicates), and phylogenetic analysis was performed using MEGA6 software (Hall 2013). The exon/intron structures of the CBS genes were determined by comparing the coding sequences and corresponding genomic sequences in the gene structure display server (GSDS, https://gsds.cbi.pku.edu.cn/) (Guo et al. 2007). The protein transmembrane topology was predicted using TMHMM Server v2.0, and tertiary protein structures were predicted using Phyre. Domain architecture was analyzed by SMART (a Simple Modular Architecture Research Tool).

### Plant Materials and Treatments

For low nitrogen treatment, seeds of a low N-tolerant soybean variety (Pohuang) were germinated. After 7 days, the seedlings were grown hydroponically in half-strength modified Hoagland solution until the first trifoliate leaf was fully developed and then grown in normal nitrogen solution (2 mM Ca(NO_3_)_2_·4H_2_O, 2.5 mM KNO_3_, 0.5 mM NH_4_NO_3_, 0.5 mM KH_2_PO_4_, 1 mM MgSO_4_·7H_2_O, 0.05 mM Fe-EDTA, 0.005 mM KI, 0.1 mM H_3_BO_3_, 0.1 mM MnSO_4_·H_2_O, 0.03 mM ZnSO_4_·7H_2_O, 0.0001 mM CuSO_4_·5H_2_O, 0.001 mM Na_2_MO_4_·2H_2_O, 0.0001 mM CoCl_2_·6H_2_O) or low nitrogen solution (0.2 mM Ca(NO_3_)_2_·4H_2_O, 1.8 mM CaCl_2_·2H_2_O, 0.25 mM KNO_3_, 1.125 mM K_2_SO_4_, 0.05 mM NH_4_NO_3_, 0.5 mM KH_2_PO_4_, 1 mM MgSO_4_·7H_2_O, 0.05 mM Fe-EDTA, 0.005 mM KI, 0.1 mM H_3_BO_3_, 0.1 mM MnSO_4_·H_2_O, 0.03 mM ZnSO_4_·7H_2_O, 0.0001 mM CuSO_4_·5H_2_O, 0.001 mM Na_2_MO_4_·2H_2_O, 0.0001 mM CoCl_2_·6H_2_O) at 25 °C in a chamber with a 12-h light and 12-h dark photoperiod. All treatments were performed over a continuous time course (0 h, 0.5 h, 2 h, 6 h, 12 h, and 3, 6, and 9 days). Roots, stems, and leaves from control and stress-treated plants (five plants were collected as mixed samples at each time point) were collected as samples in three biological replicates for RNA preparation, and the samples were quickly frozen in liquid nitrogen and stored at − 80 °C until use.

Soybean seeds were geminated in water at 25 °C in the dark under conditions of a 12--h light and 12-h dark photoperiod and 70% humidity. Salt, dehydration, cold, H_2_O_2_, and abscisic acid (ABA) stresses were applied to 2-week-old soybean seedlings. For salt stress, the roots of seedlings were dipped into solutions of 200 mM NaCl. For dehydration, the root systems of whole plants were placed onto filter paper with 70% humidity at room temperature for induction of a rapid drought treatment (Feng et al. 2015). For H_2_O_2_ stress, the roots of seedlings were dipped into solutions of 25 mM H_2_O_2_. For ABA treatment, soybean seedlings were sprayed with 100 μM ABA. For cold treatment, soybean seedlings were subjected to 4 °C. All stress treatments lasted from 0 to 12 h. Each treatment contained three independent replicates. At 0, 0.5, 5, and 12 h after each treatment, soybean seedlings were harvested, and five plants were collected as mixed samples at each time point, frozen in liquid nitrogen, and stored at − 80 °C until extraction of total RNA for qRT-PCR assays.

### Expression Analysis of GmCBSDUFs

Total RNA was isolated from soybean tissues using TRIzol reagent (Invitrogen) and treated with DNase I (Invitrogen) to avoid genomic DNA contamination. First-strand cDNA was synthesized using Superscript II reverse transcriptase (Invitrogen). Gene-specific primers were designed according to gene sequences using Primer 5.0 software (Table S1). The quantitative RT-PCR was performed with a CFX96TM real-time system (Bio-Rad) in a 20 μl system containing 2 μl of a tenfold diluted cDNA, 10 μl of 2 × SYBR green real-time PCR master mix (Takara), and 1 μl each of 10 μM forward and reverse primers. β-actin was used as the internal control. Statistical analyses were performed using the *t*-test, and *p* < 0.05 and < 0.01 were considered significant and extremely significant differences, respectively.

### Vector Construction, Arabidopsis Transformation, and Stress Treatment

The full-length coding sequence (the primers 5′ ATGGCGGCAGAGATACCG 3′ and 5′ CTATTGATTCCTTAGTGACTCACT 3′.) of GmCBSDUF3 was TA cloned into the plant expression vector pCXSN. The recombinant construct containing the 35S::GmCBSDUF3 (Fig. S2A) cassette was introduced into *Agrobacterium tumefaciens* strain GV3101 and then transformed into Arabidopsis (Columbia) via the floral dip method. The transgenic plants were screened on MS medium with 100 mg/L hygromycin and confirmed by PCR analyses. The expression levels of GmCBSDUF3 in transgenic plants were determined by qPCR (Fig. S2B).

Seeds of transgenic overexpressing Arabidopsis and WT plants were grown on 10 × 10 cm MS agar plates. They were routinely kept for 2 days in darkness at 4 °C to break dormancy and transferred in a light growth chamber under a day/night 16/8 h cycle at 23 °C. For stress treatment, the seeds of transgenic lines or WT were kept on MS media supplemented with 50 mM NaCl, 2% PEG, or 1.5 μM ABA. Each treatment contained three independent replicates.

## Electronic supplementary material

Below is the link to the electronic supplementary material.

Supplementary file1 (TIF 531 kb)—Figure S1. Transmembrane topology analysis of soybean CBSDUF proteins. The protein transmembrane topology was predicted by using TMHMM Server v2.0. The predicted transmembrane helixes are shown as red peaks.

Supplementary file2 (TIF 132 kb)—Figure S2. Generation and RT-PCR testing of GmCBSDUF3 lines (A) GmCBSDUF3 overexpression plasmid profile; (B) relative expression levels of GmCBSDUF3 lines.

Supplementary file3 (TIF 98 kb)—Figure S3. Tissue-specific expression patterns of GmCBSDUF genes. The figure shows the gene expression patterns of GmCBSDUF genes in 17 soybean GeneAtlas tissue samples, including flower (open and unopened), lateral root (standard), leaf (ammonia, nitrate, urea, standard and symbiotic condition), nodule (symbiotic condition), root tip (standard), root (ammonia, nitrate, urea, standard and symbiotic condition), shoot tip (standard), stem (standard) and 9 soybeannormal tissue samples (flower, leaf, nodule, pod, root, root hair, seed, SAM and stem). The data were obtained from the soybean genome database Phytozome 12.

Supplementary file4 (TIF 572 kb)—Figure S4. Expression of soybean GmCBSDUF genes in response to low nitrogen stresses.A: Leaves, B: stems, C: roots.Data were obtained by real-time PCR normalized against the reference gene ACT11 and are shown as apercentage of expression in the control at 0 h. Blue columns represent the expression under normalnitrogen conditions, and red columns represent the expression under low nitrogen conditions. GmCBSDUF13, which was not expressed in soybean roots, stems and leaves under normal conditions,was not induced under low nitrogen stress and is not shown in this figure.

Supplementary file5 (XLSX 10 kb)—Table S1. Primer list

Supplementary file6 (XLSX 15 kb)—Table S2. Responses of 18 soybean CBSDUF genes to abiotic stresses by qRT-PCR with the 2-ΔΔCT method

## References

[CR1] Bateman A (1997). The structure of a domain common to archaebacteria and the homocystinuria disease protein. Trends Biochem Sci.

[CR2] Brenna A, Talora C (2019). WC-1 and the proximal GATA sequence mediate a Cis-/trans- acting repressive regulation of light-dependent gene transcription in the dark. Int J Mol Sci.

[CR3] Collart FR, Osipiuk J, Trent J, Olsen GJ, Huberman E (1996). Cloning and characterization of the gene encoding IMP dehydrogenase from *Arabidopsis thaliana*. Gene.

[CR4] Diédhiou CJ, Golldack D (2006). Salt-dependent regulation of chloride channel transcripts in rice. Plant Sci.

[CR5] Espinoza C, Medina C, Somerville S, Arce-Johnson P (2007). Senescence-associated genes induced during compatible viral interactions with grapevine and Arabidopsis. J Exp Bot.

[CR6] Estevez R, Pusch M, Ferrer-Costa C, Orozco M, Jentsch TJ (2004). Functional and structural conservation of CBS domains from CLC chloride channels. J Physiol.

[CR7] Fabro G, Di Rienzo JA, Voigt CA, Savchenko T, Dehesh K, Somerville S, Alvarez ME (2008). Genome-wide expression profiling Arabidopsis at the stage of Golovinomyces cichoracearum haustorium formation. Plant Physiol.

[CR8] Feng ZJ, Cui XY, Cui XY, Chen M, Yang GX, Ma YZ, He GY, Xu ZS (2015). The soybean GmDi19–5 interacts with GmLEA3.1 and increases sensitivity of transgenic plants to abiotic stresses. Front Plant Sci.

[CR9] Gissot L, Polge C, Jossier M, Girin T, Bouly JP, Kreis M, Thomas M (2006). AKINbetagamma contributes to SnRK1 heterotrimeric complexes and interacts with two proteins implicated in plant pathogen resistance through its KIS/GBD sequence. Plant Physiol.

[CR10] Gollery M, Harper J, Cushman J, Mittler T, Girke T, Zhu JK, Bailey-Serres J, Mittler R (2006). What makes species unique? The contribution of proteins with obscure features. Genome Biol.

[CR11] Guo AY, Zhu QH, Chen X, Luo JC (2007). GSDS: a gene structure display server. Yi Chuan.

[CR12] Hall BG (2013). Building phylogenetic trees from molecular data with MEGA. Mol Biol Evol.

[CR13] Hao Q, Shang W, Zhang C, Chen H, Chen L, Yuan S, Chen S, Zhang X, Zhou X (2016). Identification and comparative analysis of CBS domain-containing proteins in soybean (*Glycine max*) and the primary function of GmCBS21 in enhanced tolerance to low nitrogen stress. Int J Mol Sci.

[CR14] Hechenberger M, Schwappach B, Fischer WN, Frommer WB, Jentsch TJ, Steinmeyer K (1996). A family of putative chloride channels from Arabidopsis and functional complementation of a yeast strain with a CLC gene disruption. J Biol Chem.

[CR15] Kereszt A, Li D, Indrasumunar A, Nguyen CD, Nontachaiyapoom S, Kinkema M, Gresshoff PM (2007). Agrobacterium rhizogenes-mediated transformation of soybean to study root biology. Nat Protoc.

[CR16] Kumari S, Sabharwal VP, Kushwaha HR, Sopory SK, Singla-Pareek SL, Pareek A (2009). Transcriptome map for seedling stage specific salinity stress response indicates a specific set of genes as candidate for saline tolerance in *Oryza sativa *L.. Funct Integr Genom.

[CR17] Kushwaha HR, Singh AK, Sopory SK, Singla-Pareek SL, Pareek A (2009). Genome wide expression analysis of CBS domain containing proteins in *Arabidopsis thaliana* (L.) Heynh and *Oryza sativa* L. reveals their developmental and stress regulation. BMC Genom.

[CR18] Li H, Han X, Qiu W, Xu D, Wang Y, Yu M, Hu X, Zhuo R (2019). Identification and expression analysis of the GDSL esterase/lipase family genes, and the characterization of SaGLIP8 in Sedum alfredii hance under cadmium stress. Peer J.

[CR19] Libault M, Farmer A, Joshi T, Takahashi K, Langley RJ, Franklin LD, He J, Xu D, May G, Stacey G (2010). An integrated transcriptome atlas of the crop model *Glycine max*, and its use in comparative analyses in plants. Plant J.

[CR20] Liu YM, Qu JT, Zhang L, Xu XY, Wei G, Zhao ZF, Ren MZ, Cao MJ (2020). Identification and characterization of the TCA cycle genes in maize. BMC Plant Biol.

[CR21] Lv Q, Tang R, Hua L, Gao X, Li Y, Zheng H, Zhang H (2009). Cloning and molecular analyses of the *Arabidopsis thaliana* chloride channel gene family. Plant Sci.

[CR22] Narusaka Y, Nakashima K, Shinwari ZK, Sakuma Y, Furihata T, Abe H, Narusaka M, Shinozaki K, Yamaguchi-Shinozaki K (2003). Interaction between two cis-acting elements, ABRE and DRE, in ABA-dependent expression of Arabidopsis rd29A gene in response to dehydration and high-salinity stresses. Plant J.

[CR23] Sahu BB, Shaw BP (2009). Isolation, identification and expression analysis of salt-induced genes in *Suaeda maritima*, a natural halophyte, using PCR-based suppression subtractive hybridization. BMC Plant Biol.

[CR24] Schmutz J, Cannon SB, Schlueter J, Ma J, Mitros T, Nelson W, Hyten DL, Song Q, Thelen JJ, Cheng J, Xu D, Hellsten U, May GD, Yu Y, Sakurai T, Umezawa T, Bhattacharyya MK, Sandhu D, Valliyodan B, Lindquist E, Peto M, Grant D, Shu S, Goodstein D, Barry K, Futrell-Griggs M, Abernathy B, Du J, Tian Z, Zhu L, Gill N, Joshi T, Libault M, Sethuraman A, Zhang XC, Shinozaki K, Nguyen HT, Wing RA, Cregan P, Specht J, Grimwood J, Rokhsar D, Stacey G, Shoemaker RC, Jackson SA (2010). Genome sequence of the palaeopolyploid soybean. Nature.

[CR25] Singh AK, Kumar R, Pareek A, Sopory SK, Singla-Pareek SL (2012). Overexpression of rice CBS domain containing protein improves salinity, oxidative, and heavy metal tolerance in transgenic tobacco. Mol Biotechnol.

[CR26] Sinharoy S, Liu C (2016). A *Medicago truncatula *cystathionine-beta-synthase-like domain-containing protein is required for rhizobial infection and symbiotic nitrogen fixation. Plant Physiol.

[CR27] Song W, Wang F, Chen L, Ma R, Zuo X, Cao A, Xie S, Chen X, Jin X, Li H (2019). GhVTC1, the key gene for ascorbate biosynthesis in cell elongation under control of ethylene. Cells.

[CR28] Thompson JD, Gibson TJ, Plewniak F, Jeanmougin F, Higgins DG (1997). The CLUSTAL_X windows interface: flexible strategies for multiple sequence alignment aided by quality analysis tools. Nucleic Acids Res.

[CR30] Wang X, Ren X, Zhu L, He G (2004). OsBi1, a rice gene, encodes a novel protein with a CBS-like domain and its expression is induced in responses to herbivore feeding. Plant Sci.

[CR29] Wang Q, Lai T, Qin G, Tian S (2009). Response of jujube fruits to exogenous oxalic acid treatment based on proteomic analysis. Plant Cell Physiol.

[CR31] Wu C, Zheng C, Ji G, Jiang P (2019). Synergistic effects of HSE and LTR elements from hsp70 gene promoter of Ulva prolifera (Ulvophyceae, Chlorophyta) upon temperature induction1. J Phycol.

[CR32] Xin S, Tao C, Li H (2016). Cloning and functional analysis of the promoter of an Ascorbate Oxidase gene from *Gossypium hirsutum*. PLoS ONE.

[CR33] Xu Z, Wang M, Guo Z, Zhu X, Xia Z (2019). Identification of a 119-bp promoter of the maize sulfite oxidase gene(ZmSO) that confers high-level gene expression and ABA or drought inducibility in transgenic plants. Int J Mol Sci.

[CR34] Yu TF, Zhao WY, Fu JD, Liu YW, Chen M, Zhou YB, Ma YZ, Xu ZS, Xi YJ (2018). Genome-wide analysis of CDPK family in foxtail millet and determination of SiCDPK24 Functions in drought stress. Front Plant Sci.

[CR35] Zhang RX, Qin LJ, Zhao DG (2017). Overexpression of the OsIMP gene increases the accumulation of inositol and confers enhanced cold tolerance in tobacco through modulation of the antioxidant enzymes activities. Genes (Basel).

