# Supplementary figures and images for: Genome-Wide Investigation and Expression Profiling Under Abiotic Stresses of a Soybean Unknown Function (DUF21) and Cystathionine-β-Synthase (CBS) Domain-Containing Protein Family
Source: Biochem Genet. 2020 Aug 10;59(1):83–113. doi: 10.1007/s10528-020-09991-w (PMC7846513; doi:10.1007/s10528-020-09991-w)

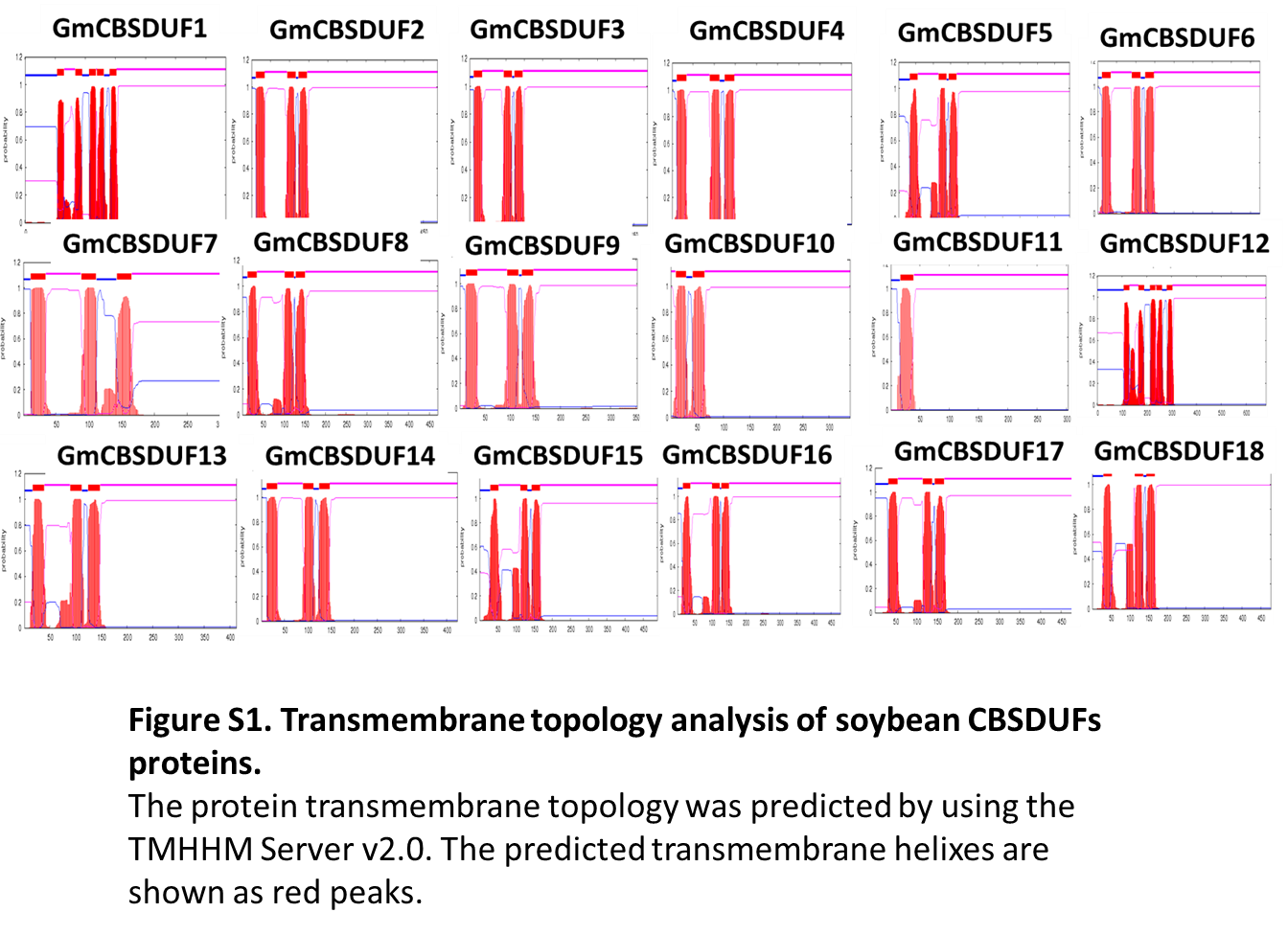

Supplement: Supplementary file 1 — Supplementary file1 (TIF 531 kb)—Figure S1. Transmembrane topology analysis of soybean CBSDUF proteins. The protein transmembrane topology was predicted by using TMHMM Server v2.0. The predicted transmembrane helixes are shown as red peaks. [file 10528_2020_9991_MOESM1_ESM.tif]

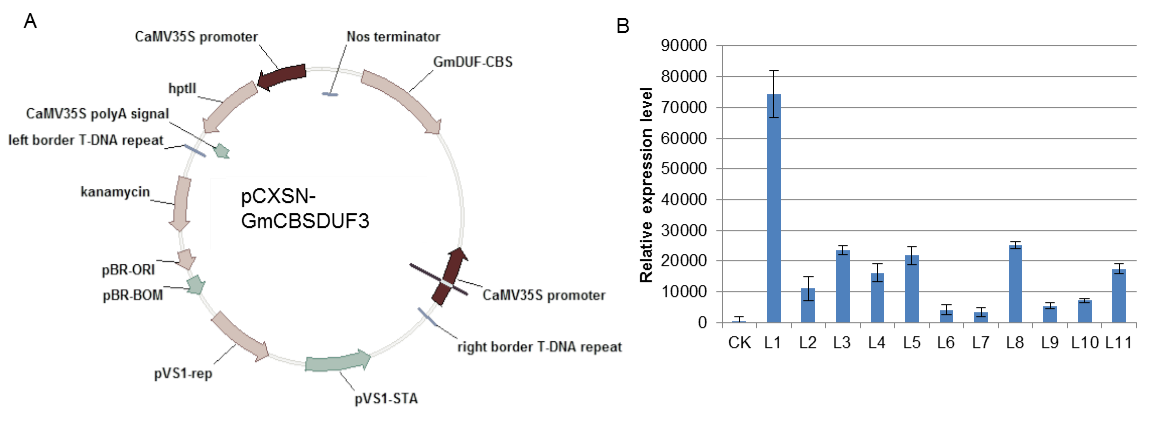

Supplement: Supplementary file 2 — Supplementary file2 (TIF 132 kb)—Figure S2. Generation and RT-PCR testing of GmCBSDUF3 lines (A) GmCBSDUF3 overexpression plasmid profile; (B) relative expression levels of GmCBSDUF3 lines. [file 10528_2020_9991_MOESM2_ESM.tif]

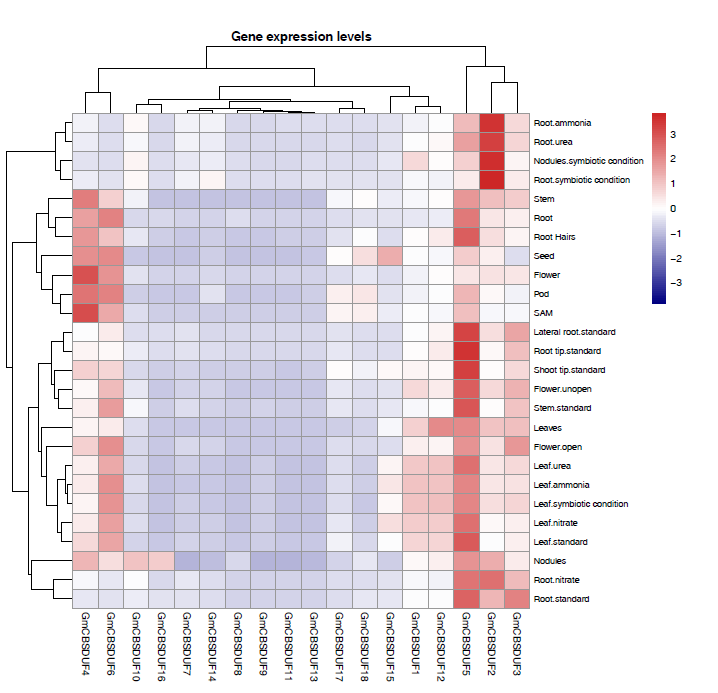

Supplement: Supplementary file 3 — Supplementary file3 (TIF 98 kb)—Figure S3. Tissue-specific expression patterns of GmCBSDUF genes. The figure shows the gene expression patterns of GmCBSDUF genes in 17 soybean GeneAtlas tissue samples, including flower (open and unopened), lateral root (standard), leaf (ammonia, nitrate, urea, standard and symbiotic condition), nodule (symbiotic condition), root tip (standard), root (ammonia, nitrate, urea, standard and symbiotic condition), shoot tip (standard), stem (standard) and 9 soybeannormal tissue samples (flower, leaf, nodule, pod, root, root hair, seed, SAM and stem). The data were obtained from the soybean genome database Phytozome 12. [file 10528_2020_9991_MOESM3_ESM.tif]

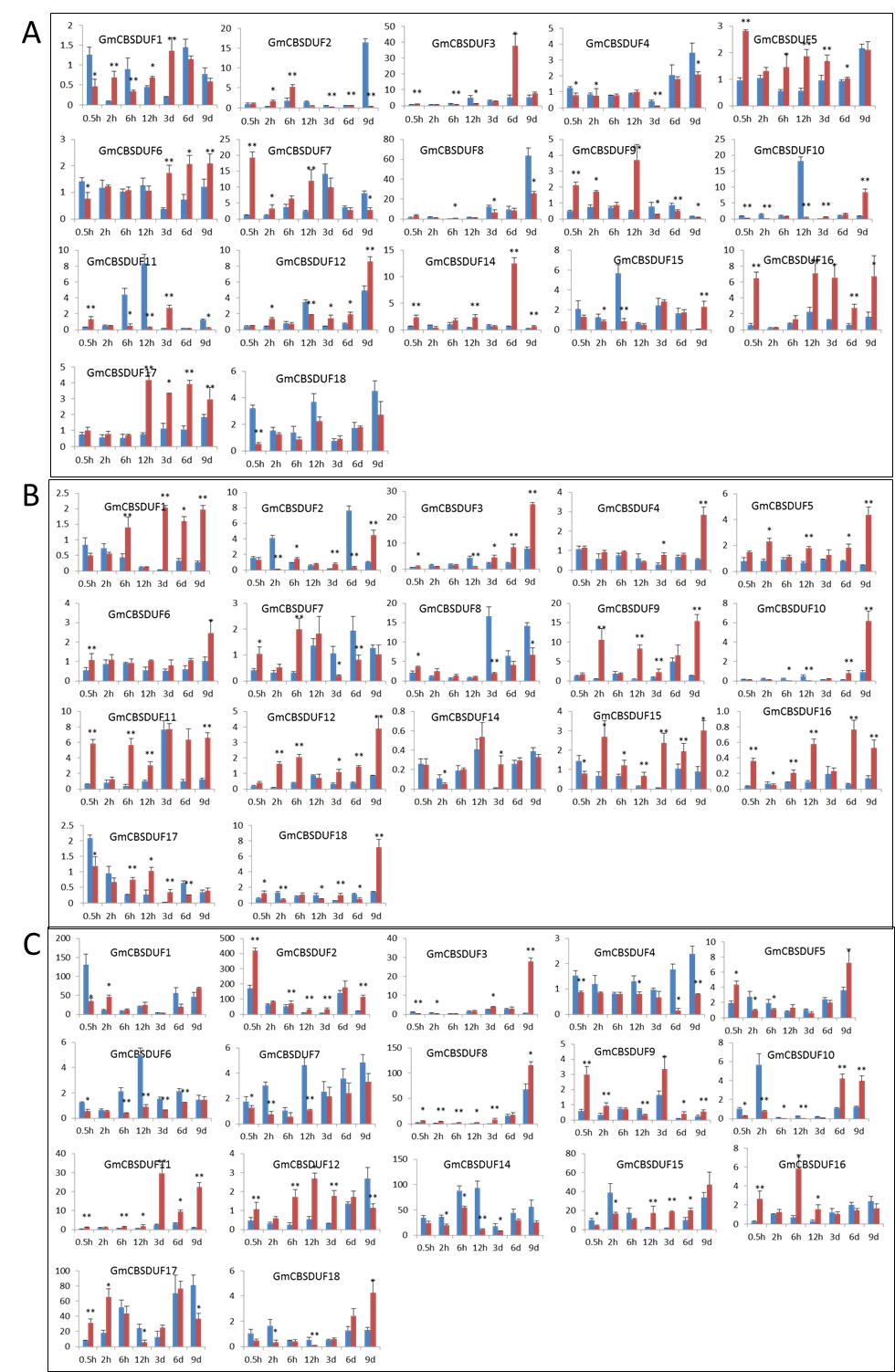

Supplement: Supplementary file 4 — Supplementary file4 (TIF 572 kb)—Figure S4. Expression of soybean GmCBSDUF genes in response to low nitrogen stresses.A: Leaves, B: stems, C: roots.Data were obtained by real-time PCR normalized against the reference gene ACT11 and are shown as apercentage of expression in the control at 0 h. Blue columns represent the expression under normalnitrogen conditions, and red columns represent the expression under low nitrogen conditions. GmCBSDUF13, which was not expressed in soybean roots, stems and leaves under normal conditions,was not induced under low nitrogen stress and is not shown in this figure. [file 10528_2020_9991_MOESM4_ESM.tif]
